# The Thylakoid Membrane Protein CGL160 Supports CF_1_CF_0_ ATP Synthase Accumulation in *Arabidopsis thaliana*

**DOI:** 10.1371/journal.pone.0121658

**Published:** 2015-04-02

**Authors:** Rikard Fristedt, Nádia Figueira Martins, Daniela Strenkert, Cornelia A. Clarke, Monika Suchoszek, Wolfram Thiele, Mark Aurel Schöttler, Sabeeha S. Merchant

**Affiliations:** 1 Department of Chemistry and Biochemistry, UCLA, Los Angeles, California, United States of America; 2 Max Planck Institute of Molecular Plant Physiology, Potsdam, Germany; 3 Institute for Genomics and Proteomics UCLA, Los Angeles, California, United States of America; University of Hyderabad, INDIA

## Abstract

The biogenesis of the major thylakoid protein complexes of the photosynthetic apparatus requires auxiliary proteins supporting individual assembly steps. Here, we identify a plant lineage specific gene, *CGL160*, whose homolog, *atp1*, co-occurs with ATP synthase subunits in an operon-like arrangement in many cyanobacteria. *Arabidopsis thaliana* T-DNA insertion mutants, which no longer accumulate the nucleus-encoded CGL160 protein, accumulate less than 25% of wild-type levels of the chloroplast ATP synthase. Severe cosmetic or growth phenotypes result under either short day or fluctuating light growth conditions, respectively, but this is ameliorated under long day constant light growth conditions where the growth, ATP synthase activity and photosynthetic electron transport of the mutants are less affected. Accumulation of other photosynthetic complexes is largely unaffected in *cgl160* mutants, suggesting that CGL160 is a specific assembly or stability factor for the CF_1_CF_0_ complex. CGL160 is not found in the mature assembled complex but it does interact specifically with subunits of ATP synthase, predominantly those in the extrinsic CF_1_ sub-complex. We suggest therefore that it may facilitate the assembly of CF_1_ into the holocomplex.

## Introduction

Oxygenic photosynthesis is catalyzed by four large protein complexes embedded in the thylakoid membrane [1]. Photosystem II (PSII) catalyzes the first step of linear electron transport, oxidizing water on the lumenal site of the thylakoid membrane and reducing plastoquinone on the stromal site. Per water molecule oxidized, two protons are released into the thylakoid lumen, while plastoquinone reduction is coupled to the uptake of two protons from the stroma. These protons are released into the thylakoid lumen during plastoquinol reoxidation at the cytochrome *b*_6_ *f* complex, which is the rate-limiting step of linear electron flux [2, 3]. When plastoquinol is reoxidized, the first electron is directly transferred via the Rieske 2Fe2S protein and cytochrome *f* to the lumenal redox carrier plastocyanin and ultimately towards PSI, where it reduces the photo-oxidized reaction center chlorophyll *a* dimer, P_700_. From P_700_, with the next light-induced charge separation, the electron is transferred via ferredoxin to NADP^+^, providing reducing power for the Calvin cycle and other reactions of primary metabolism. The second electron is transferred from the plastosemiquinone via cytochrome *b*_*6*_ to the stromal plastoquinone binding site of the cytochrome *b*_6_ *f* complex [4]. The oxidation of a second plastoquinol molecule on the lumenal site of the cytochrome *b*_6_ *f* complex provides the second electron required for full reduction of the semiquinone on the stromal site to plastoquinol, which is again coupled to proton uptake from the stroma. The fully reduced plastoquinol detaches from the stromal binding side and diffuses to the lumenal site, where it is oxidized. Thus, per electron pair abstracted from water, a total of six protons are released into the thylakoid lumen.

The ATP needed by the Calvin-Benson cycle is produced by CF_1_-CF_0_-ATP synthase, which consumes the proton motive force (pmf) established across the thylakoid membrane to catalyze the formation of ATP from ADP and orthophosphate (P_i_). The membrane-extrinsic catalytic head of ATP synthase, CF_1_, is composed of five different subunits α, β, γ, δ and ε in the stoichiometry α_3_β_3_γδε. The α_3_β_3_ hexamer forms three nucleotide-binding catalytic centers, which undergo sequential changes in their conformation, which drive ATP synthesis [5]. The conformational changes are triggered by the 360° rotation of the γ-subunit, relative to the catalytic hexamer. CF_0_ is composed of four different subunits b, b’, c and a. While a, b and b’ together form the peripheral stalk of the ATP synthase, 14 c subunits form a ring structure in the thylakoid membrane. 14 subsequent protonation events of the c ring result in a complete 360° rotation, relative to the stalk subunits. This rotation is transduced to the catalytic α_3_β_3_ hexamer via the γ-subunit, which is bound to the c_14_ ring, thus triggering the conformational changes of the catalytic hexamer, which drive the synthesis of three molecules of ATP [6].

While in the respiratory electron transport chains of bacteria and mitochondria, the vast majority of the pmf is stored as electric field component (ΔΨ), in thylakoid membranes, the ΔpH component can account for 50 to 80% of the total pmf [7, 8]. Normally, the thylakoid lumen pH value is maintained between 7.0 and 6.5, which is sufficient to drive ATP synthesis [8]. However, when photosynthetic ATP production exceeds its metabolic consumption, so that the availability of ADP and especially P_i_ decreases and ATP synthase is substrate-limited [9], the lumen pH value may drop below 6.5. This activates the lumenal enzyme violaxanthin deepoxidase, which converts the accessory pigment violaxanthin into zeaxanthin. Also, two glutamate residues on the lumenal side of the PsbS protein get protonated [10]. Together, these processes result in non-photochemical quenching (qN), the harmless thermal dissipation of excess excitation energy in the PSII antenna bed [11]. Also, a lumen pH value below 6.5 slows down plastoquinol reoxidation at the cytochrome *b*_6_ *f* complex, because protons need to be pumped against a steeper pmf (photosynthetic control) [12, 13]. Thus, proton influx into the lumen decreases and is rebalanced to the consumption of the pmf by ATP synthase.

Because ATP synthase is the key regulator of the photosynthetic proton circuit and controls the onset of photoprotective mechanisms, its abundance and activity need to be closely adjusted to the capacity of linear electron flux and to the metabolic consumption of ATP and NADPH, so that under non-stressed conditions, the pmf is sufficient to drive ATP synthesis, while non-photochemical quenching and photosynthetic control of linear electron flux are not yet activated. Therefore, ATP synthase content changes in parallel with the cytochrome *b*_6_ *f* complex, thus adjusting linear electron flux to ATP synthesis capacity [14]. As a consequence, large changes in ATP synthase abundance and activity occur during the acclimation to different environmental conditions and during leaf ontogenesis (recently reviewed by [3]). With increasing light intensity, strong increases in ATP synthase content and activity have been observed [15]. When leaf assimilation capacity decreases during leaf ontogenesis and senescence, ATP synthase and cytochrome *b*_6_ *f* complex contents decrease in parallel down to less than 20% of the level observed in young leaves [16].

In mutants suffering from a massive loss of chloroplast ATP synthase, the pmf across the thylakoid membrane is strongly increased already at low electron flux rates, qN is rapidly activated, and linear electron flux is repressed by photosynthetic control [17, 18]. However, changes in ATP synthase content do not strictly correlate with changes in its activity: in tobacco, an up to 50% repression of ATP synthase content does not result in any loss of enzyme activity, because a large fraction of ATP synthase is not fully active under standard growth conditions, so that the loss of enzymes can be compensated for by the reactivation of the inactive fraction [18].

Despite its key role in the regulation of photosynthetic light reactions and the large adjustments of its contents during plant development, not much is known about ATP synthase biogenesis or maintenance mechanisms. For the other photosynthetic complexes, the sequence of the assembly process is much better characterized, and multiple auxiliary proteins involved in subunit assembly and cofactor insertion have been identified, as reviewed by [19–22]. Phylogenomics is one approach to identify proteins functioning in the context of oxygenic photosynthesis, because important factors involved in the biogenesis of ATP synthase and other photosynthetic complexes should be conserved in all organisms performing oxygenic photosynthesis, from cyanobacteria to higher plants [23, 24]. Here, we report the characterization of a green lineage specific protein called CGL160 (Conserved Green Lineage). *CGL160* is encoded in an operon with genes for ATP synthase subunits in many cyanobacterial genomes [25]. Reverse genetic analysis in *Arabidopsis thaliana* (Arabidopsis) showed that loss of CGL160 function leads to reduction of ATP synthase levels and a growth phenotype in a fluctuating light situation where adjustment of ATP synthase levels is necessitated. Since CGL160 interacts with components of the CF_1_ sub-complex, we suggest that the protein may facilitate ATP synthase assembly.

## Materials and Methods

### Plant Growth Conditions and Mutant Characterization

Arabidopsis Columbia (Col-4) wild-type plants were obtained from the Arabidopsis Biological Resource Center (ABRC). Two T-DNA insertion mutants for Arabidopsis *CGL160* (gene ID At2g31040) were obtained: SALK_057229 (later on called *cgl160-1*) and WiscDsLoxHs024_02B (later on called *cgl160-2*). Plants were grown on soil under long day conditions (16 h light, 8 h dark) and under short-day conditions (8 h light, 16 h dark) at 120 μmol photons m^-2^ s^-1^ in a Conviron growth chamber (model MTR26) equipped with light bulbs from Philips (Master TL-D 58W/840). For fluctuating low/normal light growth conditions, plants were illuminated for 5 min with 120 μmol photons m^-2^ s^-1^, followed by 5 min of illumination with 20 μmol photons m^-2^ s^-1^. This pattern was repeated during the complete 16 hours photoperiod. The day temperature was set to 22°C, night temperature was 16°C. Relative humidity was 60% during the day and 75% during the night. Mutant plants were screened by PCR for heterozygosity or homozygosity of the T-DNA insertion using primers 057229_Forward and 057229_Reverse for SALK_057229, primers WiscHs024_Forward and WiscHs024_Reverse for WiscDsLoxHs024_02B and T-DNA left border primers LBb1 and WiscDsLoxHS for SALK and Wisc lines, respectively. DNA fragments were amplified by 40 cycles of denaturation at 94°C for 1 min, annealing at 54°C for 55 s and polymerization at 72°C for 1 min followed by a final extension for 10 min at 72°C (S1 Table).

### Complementation of cgl160-1

A cDNA fragment corresponding to *CGL160* was amplified from a cDNA clone (SALK_G13452) using a high-fidelity polymerase (Phusion; New England Biolabs. Primers used, CGL160_cloning _1-F and CGL160_cloning _1-R (S1 Table). The PCR product was subcloned into a pEGAD vector, generating a translational fusion. The resulting plasmid pEGAD-CGL160 was transformed into *Agrobacterium tumefaciens* strain AGL0 by electroporation using standard protocols. Homozygous *cgl160-1* plants were transformed by the floral dip method [26]. Transformants were selected in soil for Basta resistance (120 mg L^-1^; Bayer Scientific; Chemical Abstract Service, CAS no. 77182-82-2).

### Chlorophyll a fluorescence

Chlorophyll *a* fluorescence of intact leaves was measured at 22°C using the fiber version of the Dual-PAM instrument (Walz GmbH, Effeltrich, Germany). Light response curves of linear electron flux, non-photochemical quenching (qN) and of the redox state of the PSII acceptor side (qL) were measured after 30 min of dark adaptation. Then, the light intensity was step-wise increased from 0 to 1000 μE m^-2^ s^-1^, with a measuring time of 150 s per light intensity. The 77 K chlorophyll *a* fluorescence emission spectra of freshly isolated thylakoids equivalent to 10 μg chlorophyll ml^-1^ were measured using an F-6500 fluorometer (Jasco GmbH, Groß-Umstadt, Germany). The sample was excited at 430 nm wavelengths using a 10 nm bandwidth, and the emission spectrum between 660 and 800 nm wavelengths was recorded with a bandwidth of 1 nm. The spectra were corrected for the instrumental response.

### Pmf measurements and ATP synthase activity

The electrochromic shift signal (ECS) was used as a non-invasive probe to determine the pmf across the thylakoid membrane, its partitioning, and ATP synthase activity in intact Arabidopsis leaves. Signals were measured and deconvoluted as described [18]. The maximum amplitude of the ECS (ECS_T_) was used as a measure for the maximum light-induced pmf across the thylakoid membrane. The measurements were performed at 22°C, and leaves were illuminated for 10 min prior to each measurement with saturating light (1400 μmol photons m^-2^ s^-1^) to fully activate the Calvin cycle and ensure that photosynthesis was in steady state. The saturating illumination was then interrupted by 15 s intervals of darkness, and the dark-interval relaxation of the ECS was measured. The rapid first phase of the ECS decay kinetic was fitted with a single exponential decay function. The reciprocal value of the lifetime of the ECS decay kinetic, the thylakoid conductivity for protons (gH^+^) was used as a measure of ATP synthase activity, because this rapid ECS decay kinetic is exclusively attributable to proton efflux through chloroplast ATP synthase [27]. In case of the wild type, the first 150 ms of the ECS decay kinetic were analyzed, while in case of the mutants, the first 250 to 300 ms of the dark interval were fitted. Pmf partitioning into ΔpH and ΔΨ was determined by analyzing the slowly relaxing phase of the ECS between 1 and 15 s in darkness [18].

### Thylakoid membrane isolation and quantitation of photosynthetic complexes

Thylakoid membranes were isolated according to [28, 29]. The contents of PSII and the cytochrome *b*_6_ *f* complex were determined from difference absorbance signals of cytochrome *b*_559_ (PSII) and cytochromes *b*_6_ and *f* in destacked thylakoids equivalent to 50 μg chlorophyll ml^-1^. All cytochromes were fully oxidized by the addition of 1 mM potassium hexacyanoferrate (III), and then step-wise reduced by the addition of 10 mM sodium ascorbate, which is sufficient to reduce the high-potential form of cytochrome *b*_559_ and cytochrome *f*, and of 10 mM sodium dithionite, which fully reduces the low potential form of cytochrome *b*_559_ and the two b-type hemes non-covalently bound to cytochrome *b*_*6*_. Using a V-550 spectrophotometer equipped with a head-on photomultiplier (Jasco GmbH, Groß-Umstadt, Germany), at each of the three redox potentials, absorbance spectra were measured between 575 and 540 nm wavelength. The scanning speed was 100 nm / min, and a spectral bandwidth of 1 nm was selected. To improve the signal-to-noise ratio, ten spectra per redox condition were averaged. Difference spectra were calculated by subtracting the spectrum measured in the presence of 1mM potassium hexacyanoferrate from the spectrum measured in the presence of 10 mM sodium ascorbate, and by subtracting the sodium ascorbate spectrum from the spectrum measured in the presence of sodium dithionite, respectively. Finally, a baseline calculated between 540 and 575 nm wavelength was subtracted from the signals. Then, the difference spectra were deconvoluted using reference spectra as described [30, 31]. For the quantification of the cytochrome *b*_6_ *f* complex, baseline-corrected extinction coefficients of 28.0 mM^-1^ cm^-1^ and 24.6 mM^-1^ cm^-1^ were used for cytochrome *f* and each b-type heme bound to cytochrome *b*_6_, respectively. For the quantification of PSII, the sum of the difference absorbance signals of the high-potential and low-potential form of cytochrome *b*_559_ was calculated, and a baseline-corrected extinction coefficient of 20.0 mM^-1^ cm^-1^ was used.

Plastocyanin contents, relative to PSI, were determined in intact leaves and then recalculated based on the absolute PSI quantification performed in isolated thylakoids [16, 18]. PSI was quantified from light-induced difference absorbance changes of the chlorophyll-*a* dimer special pair, P_700_. Thylakoids equivalent to 50 μg chlorophyll ml^-1^ were solubilized in the presence of 0.2% (w/v) β-dodecylmaltoside in the presence of 100 μM methylviologen as artificial electron acceptor and of 10 mM sodium ascorbate as electron donor. Photooxidation was achieved by the application of a light pulse of 250 ms length (2000 μmol photons m^-2^ s^-1^). The PC-P_700_ version of the Dual-PAM instrument (Heinz Walz GmbH) was used for the measurements.

### Protein gel electrophoresis and immunoblotting

Thylakoid proteins separated by SDS-polyacrylamide gel electrophoresis (Perfect Blue twin gel system, Peqlab GmbH, Erlangen, Germany) were transferred to a polyvinylidene difluoride membrane (Hybond P) using a tank blotting system (Perfect Blue Web M, PeqLab GmbH). Specific polyclonal antibodies (produced in rabbits) against PsbB (order number: AS04 038), PSBO (AS05 092), LHCB4 (AS04 045), PetA (AS06 119), PetB (AS03 034), PETC (AS08 330), PsaB (AS10 695), PSAD (AS09 461), LHCA4 (AS01 008), AtpA (AS08 304), AtpB (AS05 085), ATPD (AS10 1591), AtpF (AS10 1604) and AtpI (AS10 1583) were all purchased from Agrisera AB (Vännäs, Sweden). As secondary antibody, an anti-rabbit IgG peroxidase conjugate was used (Sigma-Aldrich, St. Louis, USA). Immunochemical detection was carried out with the ECL Prime system (GE Healthcare, Freiburg, Germany), according to the instructions of the manufacturer, and chemiluminescence was detected using a G:Box Chemi XT4 system (Syngene, Cambridge, United Kingdom). For quantitative analysis of the chemiluminescence signals, the GeneTools software from Syngene was used.

### Subfractionation of plant cell compartments and CGL160 localization studies

For subfractionation of thylakoids, a digitonin solution of 2% (w/v) was added to the thylakoid suspension (0.6 mg chlorophyll/mL) to a final concentration of 1% (w/v). The mixture was homogenized in a glass homogenizer five times and mixed for 5 min at room temp. The solution was centrifuged at 1000*g* for 5 min to pellet unsolubilized material. The supernatant was further centrifuged at 40,000*g* for 30 min, and the stroma lamellae (non-appressed membranes) were collected from the resulting supernatant by centrifugation at 140,000*g* for 90 min. The 40,000*g* pellet contained the grana stacks (appressed membranes) [32]. Plant mitochondria were isolated from fresh organic spinach as described in [33]. The various subcellular fractions were separated by SDS-PAGE (6% stacking gel, 14% separation gel, with 6 M urea), and the proteins were subsequently transferred to polyvinylidene difluoride (PVDF) membranes (Immobilon; Millipore). An antibody specific for CGL160 was produced by immunization of rabbits with a recombinant protein corresponding to amino acids 50 to 210 of CGL160 (Agrisera AS12 1853). Antibodies against the DE-loop in the D1 protein (AS10 704), D2 (AS06 146) LHCB1 (AS01 004) and LHCA1 (AS01 005) were obtained from Agrisera. Antibodies against CF_1_ were generated by immunization with the purified CF_1_ complex from spinach [34]. PsaA specific antibody was kindly provided by Dr. Jean-David Rochaix (University of Geneva) [35], the Rubisco large subunit (RbcL) antibody was a gift from Dr. Steve Rodermel (Iowa State University) [36]. The TOM40 antibody was kindly provided by Dr. Jim Whelan (University of western Australia). Blotted membranes incubated at room temperature with horseradish peroxidaseconjugated secondary antibody for 1 h. the immunoreactive proteins were visualized following with detection reagents from the SuperSignal WestPico HRP detection kit (Thermo Scientific). Quantification of the immunoblots was done using the Fujifilm LAS-1000 software. For BN-PAGE, purified thylakoid membranes were resuspended in 20% (v/v) glycerol and 25 mM BisTris-HCl, pH 7.0, to a final chlorophyll concentration of 2 mg/mL. An equal volume of 1.5% (w/v) *n*-dodecyl β-D maltoside (DM) dissolved in resuspension buffer was added, and the mixture was incubated on ice for 10 min. After centrifugation at 14,000*g* for 30 min, the supernatant was supplemented with 0.1 volume of sample buffer (100 mM BisTris-HCl, pH 7.0, 0.5 M ε-amino-*n*-caproic acid, 30% (w/v) sucrose, 50 mg/mL Serva blue G and subjected to blue-native gel electrophoresis with a gradient of 5–13.5% acrylamide in the separation gel. The electrophoresis was performed at 4°C and 100–200 V (with an increment of 10 V every 30 min) for 5 h.

### Protein Cross-linking

Thylakoid membranes (0.5 mg chl/ml) were isolated as described and treated with newly made dithiobis succinimidyl-propionate (DSP) (Pierce) for 30 min at room temperature. The reaction was stopped by the addition of Tris-HCl, pH 8.0, to a final concentration of 50 mM and incubated on ice for 30 min. Thylakoid membranes were washed twice and finally resuspended in SDS sample buffer without *β*-mercaptoethanol. Samples were subjected to SDS-PAGE with 14% acrylamide and 6 M urea and used for immunoblotting [37]. To separate the native thylakoid protein complexes into their subunits, BN-PAGE lanes were excised and treated with denaturing sample buffer (2% (w/v) SDS, 0.8% (v/v) β-mercaptoethanol) for 30 min at room temperature in the dark and loaded on SDS-PAGE gels (12% gels containing 6 M urea). Gels were subsequently subjected to immunoblotting as described above.

### qRT-PCR

Total RNA was isolated using the RNeasy Plant MiniKit from Qiagen (74904) according to the manufacturers instructions. DNA was removed from total RNA using TURBO DNase from Ambion/ Applied Biosystems. cDNA for qRT-PCR analyses was synthesized using M-MLV reverse transcriptase from Invitrogen according to the manual in the presence of RNase inhibitor (RNasin from Promega) and by priming with random hexamers oligonucleotides. cDNA was diluted 10-fold before use. qRT-PCR reactions contained 4 μL of cDNA, 6 pmol of each forward and reverse oligonucleotide (see S1 Table), 2 μL Taq-polymerase, 0.5 μL 10 mM deoxynucleotide triphosphate (New England Biolabs), 2 μL 10x Ex Taq buffer (Mg^2+^ plus) (TaKaRa), 2 mL 10x SYBR mix (0.1% [v/v] SYBR Green 1 Nucleic Acid Gel Stain from Cambrex, 1% [v/v] Tween 20, 1 mg mL21 BSA, and 50% [v/v] DMSO) in a 20-mL volume. Each sample was analyzed in technical triplicates using DNA Engine Opticon 2 from MJ Research. The PCR program included the following steps: 95°C for 3 min, followed by 39 cycles of 95°C for 10 s, 55°C for 30 s, and 72°C for 20 s. Fluorescence was measured after each cycle at 72°C. A melting curve was performed afterwards from 70 to 95°C with plate reads every 0.5°C. Relative fold changes were calculated using the 2^-ΔΔCT^ method [38] and relative abundances were calculated according to [39]. Actin served as reference transcript. The primers used to analyze the subunits of the chloroplast ATP synthase was generated before in [40].

## Results

### The Greencut gene CGL160 encodes a protein localized to chloroplasts, which is co-expressed with structural subunits of chloroplast ATP synthase

**Fig 1:**
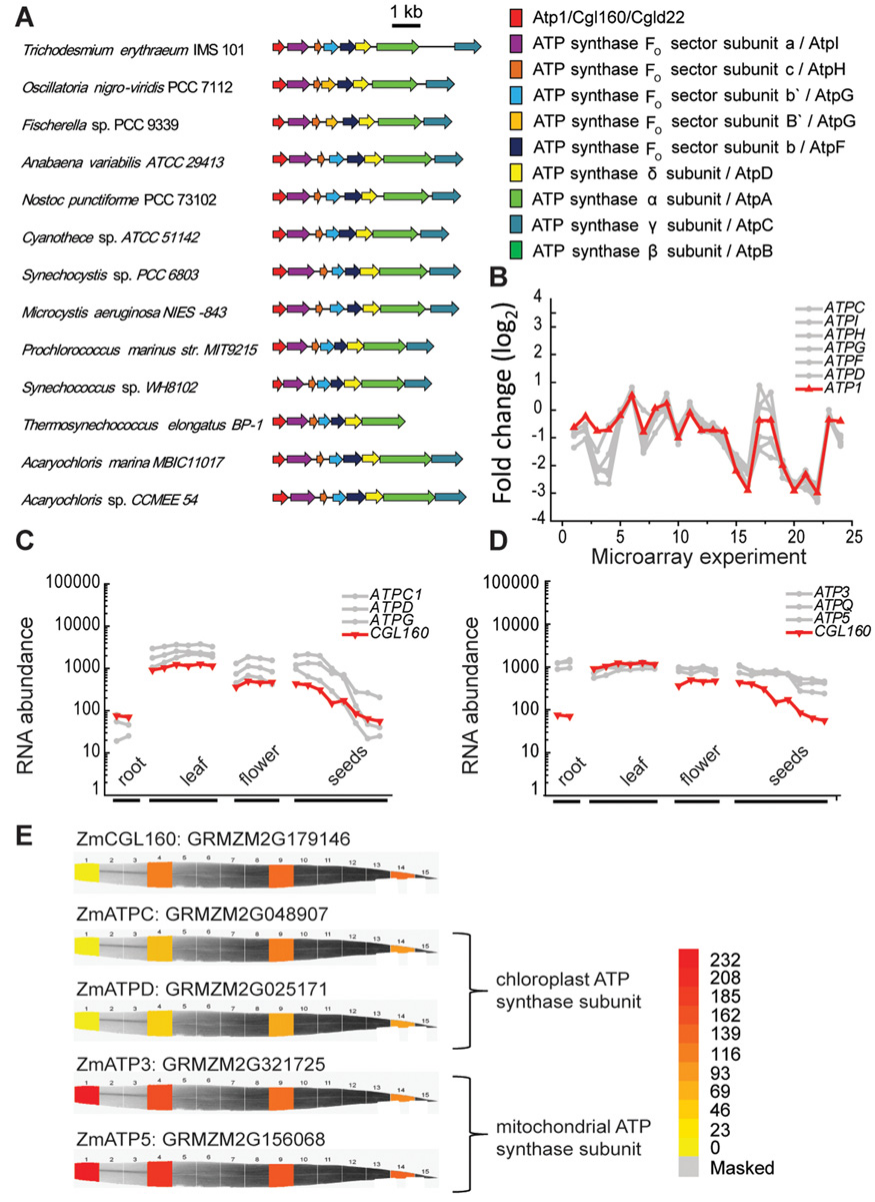
Organization of the ATP synthase operon of cyanobacteria and co-expression of genes of the ATP synthase operon with CGL160 in Synechocystis sp PCC 6803. A. The genes encoding the ATP synthase are organized in a single operon together with atp1 that is related to CGL160 (previously CGLD22 in Chlamydomonas). The genes are depicted as arrows, with the orientation indicated by the direction of the arrow. This information was obtained from The SEED viewer (http://pubseed.theseed.org) B. The genes of the ATP synthase are co-expressed with atp1 as described in [41]. The atp1 gene encodes the putative ortholog of CGL160; the curve showing the expression profile of atp1 is in red. The microarray data used to generate the expression curves were obtained from the Gene Expression Omnibus. Microarray values normalized against the median of the ratio of each sample against the reference, and log-transformed. The plotted data include 24 experiments. C-D. Affymetrix (ATH1 Gene Chip) Gene expression data in different Arabidopsis organs for CGL160 and for select nucleus-encoded genes. Genes for chloroplast localized ATP synthase subunits C, or D, mitochondrial ATP synthase. Data were retrieved from the Arabidopsis Electronic Fluorescent Pictograph (eFP) Browser. E. Expression profile of CGL160 and mitochondrial ATP synthase genes in maize (Zea mays) (ATP3, RMZM2G321725; ATP5, GRMZM2G156068) and chloroplast ATP synthase genes (ATPC, GRMZM2G048907; ATPD: GRMZM2G025171) during maize leaf development. Representative images for CGL160 and ATP synthase encoding genes are taken from http://bar.utoronto.ca/efp_maize/cgi-bin/efpWeb.cgi. Proteins that are involved in photosynthesis typically exhibit peak expression in mature chloroplasts corresponding to leaf sections 9 and 14.

We were interested in CGL160 as a candidate ATP synthase assembly or regulation factor because in previous work, when the protein was named CGLD22 in Chlamydomonas, manual curation had indicated a sequence relationship (37% sequence identity of the Chlamydomonas protein on blastp) to a protein called Atp1 in *Synechocystis* (sll1321) [41]. Although the function of Atp1 in Synechocystis is not known, its organization in the *atp* operon of *Synechocystis* sp. PCC 6803 encoding the ATP synthase gene was suggestive [41]. To validate the hypothesis concerning CGL160 function, we surveyed a total of 126 cyanobacterial genomes (S1 Fig.). We chose 13 organisms in the SEED database for analysis based on phylogenetic diversity to retrieve sequences of the *atp* operon. Indeed in all strains, *atp1* is organized in a similar operon-like arrangement together with the other *atp* genes. This is meaningful considering the diversity of the cyanobacteria chosen for the analysis (Fig. 1A, S1 Fig.).

In *Synechocystis* sp. 6803, *atp1* is co-expressed with genes encoding subunits of the ATP synthase *atpI* (sll1322), *atpH* (ssl2615), *atpG* (sll1323), *atpF* (sll1324), *atpD* (sll1325), *atpA* (sll1326), and *atpC* (sll1327), (Fig. 1B), which is compatible with co-transcription in an operon [42].

The conservation of Atp1 in the green lineage and diatoms argues in favour of its operation in the chloroplast, nevertheless there is a formal possibility that it may reside in the mitochondrion in some aspect of F_1_F_o_ function. Therefore we took advantage of proteomic and transcriptomic databases for Arabidopsis and maize to strengthen the model concerning a role in the chloroplast. In Arabidopsis, a homologue of the cyanobacterial *atp1 CGL160*, is encoded in the nucleus (At2g31040). The protein has a total length of 350 aminoacids, and according to bioinformatics predictions (http://www.uniprot.org/uniprot/O82279), there is a transit peptide for import into the chloroplast at its N-terminus. For the mature protein, a molecular mass of 33 kDa with four transmembrane domains is predicted. Co-expression analyses represent a valuable tool for investigating genes that are supposed to collaborate in a shared function. Even though At*CGL160* is expressed with the nuclear genes encoding subunits of both the chloroplast and mitochondrial ATP synthases in leaves, flowers and seeds, presumably due to the high, constitutive expression of ATP synthase genes in general, we can discriminate between the expression of genes for the chloroplast localized ATP synthase vs. the mitochondrial ones specifically by checking the expression profile in the roots of Arabidopsis (Fig. 1C and D). In the roots, *CGL160* is co-expressed with genes (*ATPC1*, *ATPD*, *ATPG)* for the chloroplast localized ATP synthase but not with mitochondrial ones (Fig. 1C and D). The same is true in maize where we find the strongest co-expression between Zm*CGL160* (GRMZM2G179146) and the genes for the chloroplast localized ATP synthase (Fig. 1E). Thus we could focus our attention on the chloroplast ATP synthase with respect to interpreting a function for CGL160.

**Fig 2:**
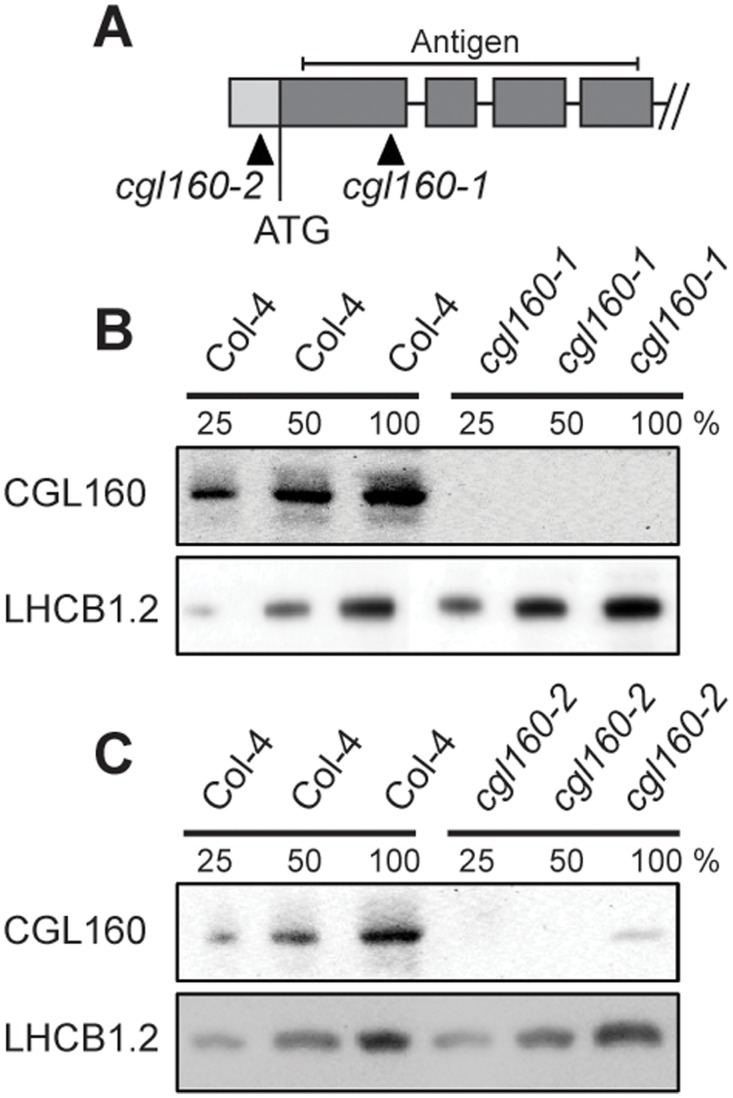
Isolation and characterization of Arabidopsis *cgl160* mutants. A. DNA insertion sites in CGL160 gene of Arabidopsis. DNA insertion sites (black triangles) are shown in relation to the CGL160 gene structure. The two *cgl160* alleles analyzed in this study are denoted as *cgl160-1* and *cgl160-2*. The CGL160 coding region is indicated by the translational start (ATG). The CGL160 genomic locus contains nine exons but only the first four are shown in the fig (grey boxes), shown are also the first four introns (black thin connecting lines). Before ATG is the promoter region in light gray. The region used for CGL160 specific antibody is shown as antigen. B. Characterization of CGL160 amount in Arabidopsis *cgl160-1* mutant from isolated chloroplasts. The CGL160 antibody was used for immunoblotting and 10 μg protein was loaded in each lane. The LHCB1 antibody was used as a loading control. C. Characterization of CGL160 amount in Arabidopsis *cgl160-2* mutant from isolated chloroplasts. The CGL160 antibody was used for immunoblotting and 10 μg protein was loaded in each lane. The LHCB1 antibody was used as a loading control.

To investigate the function of CGL160 in Arabidopsis, two *cgl160* T-DNA mutant lines were obtained from the Arabidopsis Biological Resource Centre (Ohio State University, Columbus, OH). Both mutants were characterized by PCR and found homozygous for the DNA insertion (data not shown). In *cgl160-1*, the insertion localized to exon 1 while in *cgl160-2*, it localized close to the start codon at the 5' UTR (Fig. 2A).

To determine if residual amounts of CGL160 still remain in the mutants, we generated a specific antibody by immunizing rabbits with a protein fragment covering the first four exons of CGL160 (Fig. 2A). As predicted from the sites of T-DNA insertions, *cgl160-1* is a true knockout (Fig. 2B) while in *cgl160-2* there is still some protein remaining to approximately ~20% of wild-type level (Fig. 2C).

**Fig 3:**
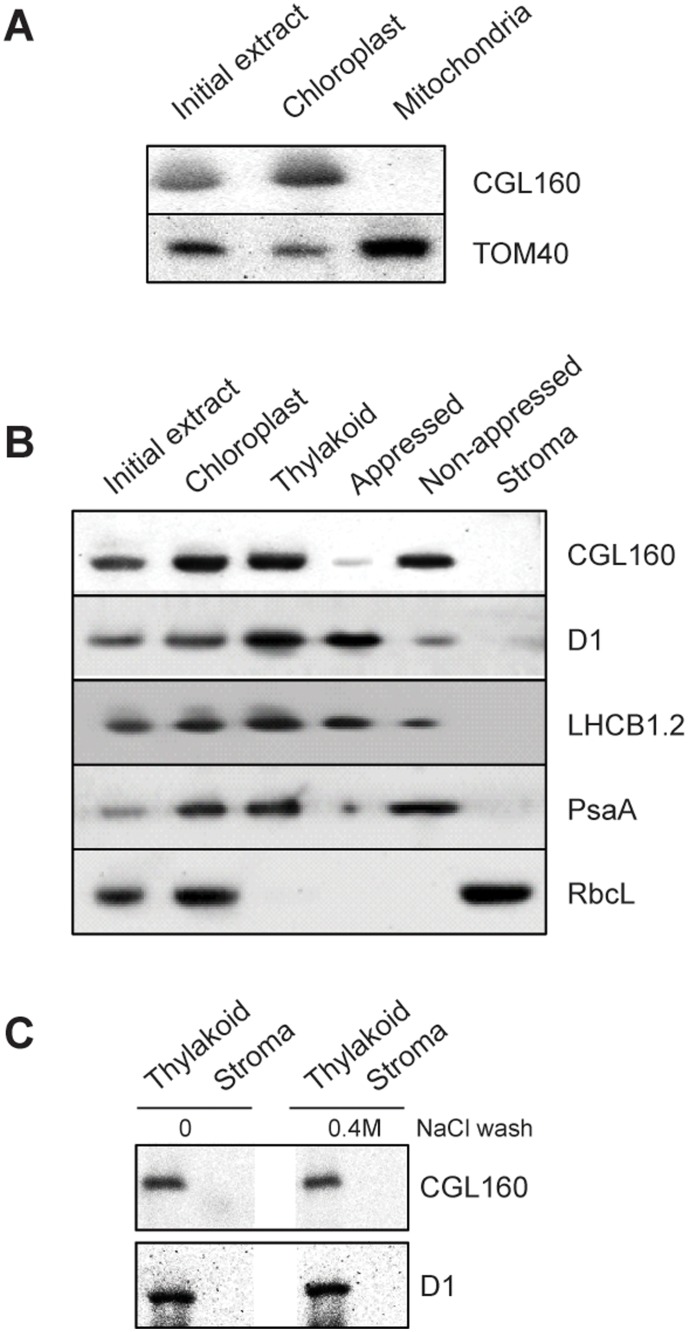
CGL160 is a membrane-integral chloroplast protein located in non-appressed thylakoid membranes. A. Subcellular localization of CGL160. Immunoblot analysis performed on initial plant extract, isolated chloroplasts or isolated mitochondria. TOM40 is a marker for the mitochondrion. B. Suborganellar localization determined by immunoblot analysis of chloroplast proteins diagnostic for photosystem I (PsaA), photosystem II (D1), Rubisco, LHCII (LHCB1.2) and CGL160. Protein extracts were separated by SDS-PAGE and probed with specific antibodies directed against PsaA, (PSI reaction center subunit), D1 (PSII reaction center subunits), LHCB1 (outer PSII antenna protein), the large subunit of Rubisco, a soluble protein in the stroma, and CGL160. C. Immunoblot analysis show that CG160 is an integral membrane protein associated with the thylakoid membranes. Isolated thylakoid membranes were washed with 0.4 M NaCl, and the thylakoid membranes and the supernatant were probed by immunoblotting with antibodies against CGL160 and the PSII reaction center protein D1.

To address the subcellular localization of CGL160 in plants, we used the specific antibody and probed various plant cell compartments (Fig. 3A and B).

When total plant protein extracts were assayed with the antibody, as expected, a single protein of approximately 32 kDa was recognized. This value is very similar to the calculated molecular mass of CGL160 if the mass of the putative transit peptide (http://www.uniprot.org/uniprot/O82279) is subtracted and is exactly the same size as the band missing in the mutant plants extracts (Fig. 2B, C). To investigate the possibility that CGL160 localizes to the mitochondria we isolated mitochondria and chloroplasts (Fig. 3A). Immunoblot experiments including the marker protein TOM40 for the mitochondria clearly revealed that CGL160 is only located to chloroplasts (Fig. 3A).

Total plant extract, chloroplasts, thylakoid membranes, appressed membranes (grana), non-appressed membranes (stroma lamellae) and soluble stroma were purified and tested with the CGL160-specific antibody (Fig. 3B). Immunoblot characterization including proper marker proteins for each compartment indicates that the CGL160 protein is enriched in chloroplasts and further to the non-appressed regions of the thylakoid membranes (Fig. 3B).

Treatment of isolated thylakoid membranes with NaCl did not remove any CGL160, indicating that CGL160 associates strongly to the thylakoid surface (Fig. 3C). As a control for the procedure, the PSII core protein D1 was assayed as well and showed the expected fractionation indicating that it is an integral membrane protein in the appressed membranes (Fig. 3C).

### CGL160 lacking plants suffer from a decreased chloroplast ATP synthase activity

**Fig 4:**
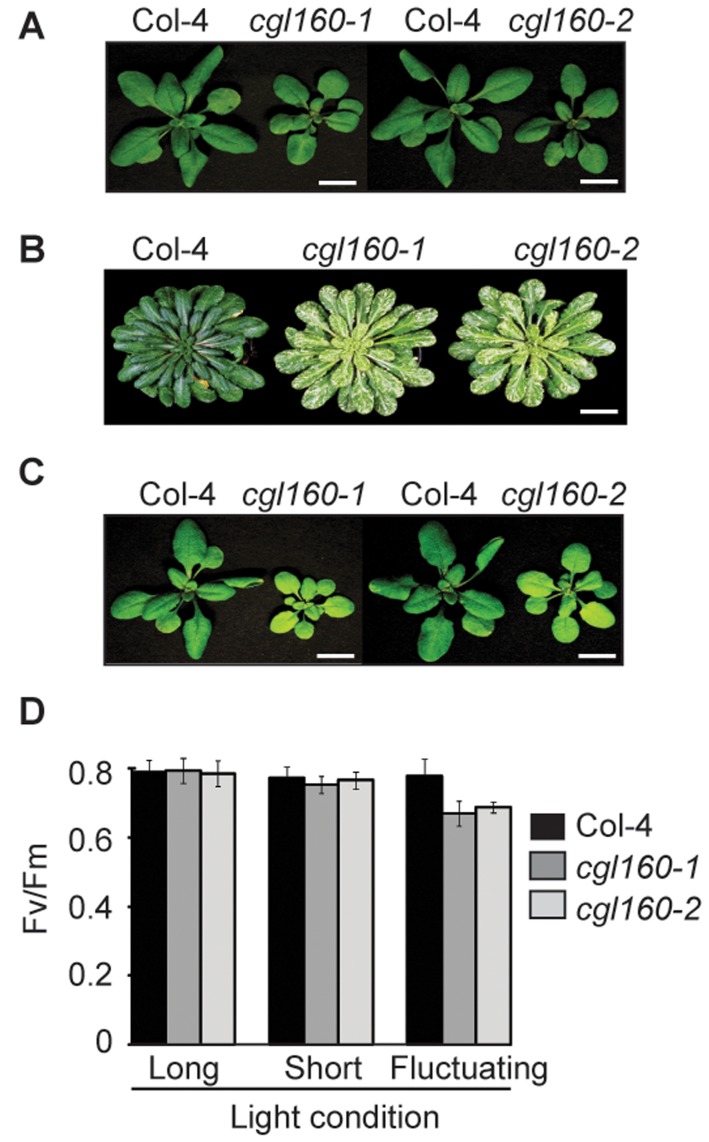
Phenotypic characterization of Arabidopsis *cgl160* mutant plants. A. Top panel shows, from left to right, a wild type plant, *cgl160-1* and wild type, *cgl160-2* plants grown for 3 weeks under normal light conditions (120 μmol m^-2^ s^-1^) at long day (16 hours light and 8 hours dark). B. Wild type, *cgl160-1* and *cgl160-2* plants grown under short day conditions (120 μmol m^-2^ s^-1^; 8 hours light and 16 hours dark) for 4 weeks. C. Shows, from left to right, a wild type plant, *cgl160-1* and wild type, *cgl160-2* plants grown for 1 week under normal light conditions (120 μmol m^-2^ s^-1^) at long day (16 hours light and 8 hours dark) and then shifted to fluctuating low/normal light conditions (5 min 120 μmol m^-2^ s^-1^, 5 min 20 μmol m^-2^ s^-1^ changing every 5 min for 16 hours light and then 8 hours dark) for two weeks. D. Bar graph of Fv/Fm as a measure of photosynthetic performance under the indicated conditions.

Because the conservation of CGL160 in oxygenic photosynthetic organisms suggested a function related to photosynthesis, and because CGL160 in cyanobacteria is encoded in the ATP synthase operon, we first analyzed photosynthetic functions with a special focus on ATP synthase-related processes in the two *cgl160* T-DNA insertion lines and in the wild type. To this end, the plants were grown under long-day (16 h day length) and short-day (8 h day length) conditions at 120 μmol m^-2^ s^-1^ light intensity. Additionally, plants were challenged with fluctuating light regimes (5 min at 120 μmol m^-2^ s^-1^, 5 min at 20 μmol m^-2^ s^-1^ changing every 5 min for a 16 hours light period). While under long-day conditions, the two CGL160 T-DNA insertion lines only showed minor growth retardation (Fig. 4A), under short-day conditions, growth was more clearly delayed, and the leaves showed a patchy phenotype (Fig. 4B).

Growth under fluctuating light resulted in pale-green mutants with small rosettes, which were massively retarded in growth (Fig. 4C). These phenotypes are supported by differences in shoot biomass accumulation, which we determined for the wild type and the *cgl160-1* mutant under long-day and fluctuating light conditions at several time points after seed germination (S2 Fig.).

Under fluctuating light conditions, the reduction in shoot biomass accumulation of the *cgl160-1* mutant was much more pronounced. Furthermore, while both under long-day and short-day conditions, the maximum quantum yield of PSII was indistinguishable between the wild type and the mutants (~0.8), under fluctuating light conditions, a significant reduction of F_V_/F_M_ was observed (~0.8 wild type and ~0.7 mutant) (Fig. 4D).

Because of the massive growth retardation under fluctuating light, we undertook detailed physiological characterization only with plants grown under constant long-day and short-day conditions. Despite the patchy phenotype under short-day conditions, the average leaf chlorophyll content was only marginally decreased relative to the wild type under both growth conditions, and also the chlorophyll *a*/*b* ratio was not statistically significantly different from wild-type plants (Table 1).

**Table 1:** Photosynthetic parameters in wild-type plants and the two *cgl160* T-DNA-insertion lines were determined for plants grown under long-day and plants grown under short-day conditions.

Parameter	Long day	Long day	Long day	Short day	Short day	Short day
Genotype	Col-4	*cgl160-1*	*cgl160-2*	Col-4	*cgl160-1*	*cgl160-2*
Number of biological replicates	**8**	**8**	**5**	**7**	**4**	**7**
Chlorophyll *a*/*b*	**3.39 ±** 0.10	**3.48 ±** 0.19	**3.43 ±** 0.21	**3.45 ±** 0.16	**3.35 ±** 0.12	**3.23 ±** 0.10
Chlorophyll / leaf area [mg m^-2^]	**456.0 ±** 42.6	**471.4 ±** 87.0	**442.2 ±** 10.8	**355.4 ±** 46.1	**337.4 ±** 44.7	**326.2 ±** 33.8
F_V_ / F_M_	**0.83 ±** 0.00	**0.83 ±** 0.01	**0.83 ±** 0.01	**0.82 ±** 0.01	**0.82 ±** 0.01	**0.82 ±** 0.01
ETR II [μmol electrons m^-2^ s^-1^]	**59.1 ±** 10.2	**45.7 ±** 4.4	**48.3 ±** 3.8	**42.1 ±** 3.2	***32*.*2 ±*** *4*.*5*	***30*.*1 ±*** *3*.*7*
Photosystem II [μmol m^-2^]	**1.09 ±** 0.17	**1.17 ±** 0.31	**1.11 ±** 0.13	**0.90 ±** 0.14	**0.90 ±** 0.08	**0.85 ±** 0.10
Cytochrome *b*_6_ *f* complex [μmol m^-2^]	**0.42 ±** 0.07	**0.40 ±** 0.12	**0.40 ±** 0.03	**0.32 ±** 0.05	**0.32 ±** 0.04	**0.27 ±** 0.04
Plastocyanin [μmol m^-2^]	**2.99 ±** 0.34	**3.03 ±** 0.17	**2.74 ±** 0.35	**2.12 ±** 0.21	**2.05 ±** 0.31	**1.82 ±** 0.33
Photosystem I [μmol m^-2^]	**1.00 ±** 0.13	**1.02 ±** 0.17	**0.91 ±** 0.04	**0.75 ±** 0.07	***0*.*65 ±*** *0*.*09*	***0*.*62 ±*** *0*.*07*

Chlorophyll *a*/*b* ratio, chlorophyll content per leaf area, maximum quantum efficiency of PSII in the dark-adapted state, and maximum linear electron flux capacity, as determined from the yield of PSII, were determined on intact leaves. Photosynthetic complex contents were determined in isolated thylakoids, and re-normalized to a leaf area basis. The values represent averages of four to eight plants, the standard deviations are indicated. Data were subjected to a one-way analysis of variance (ANOVA) using a pair-wise multiple comparison procedure (Holm-Sidak method). Statistically significant differences, relative to the wild type grown under the corresponding growth regime, are shown in italics.

**Fig 5:**
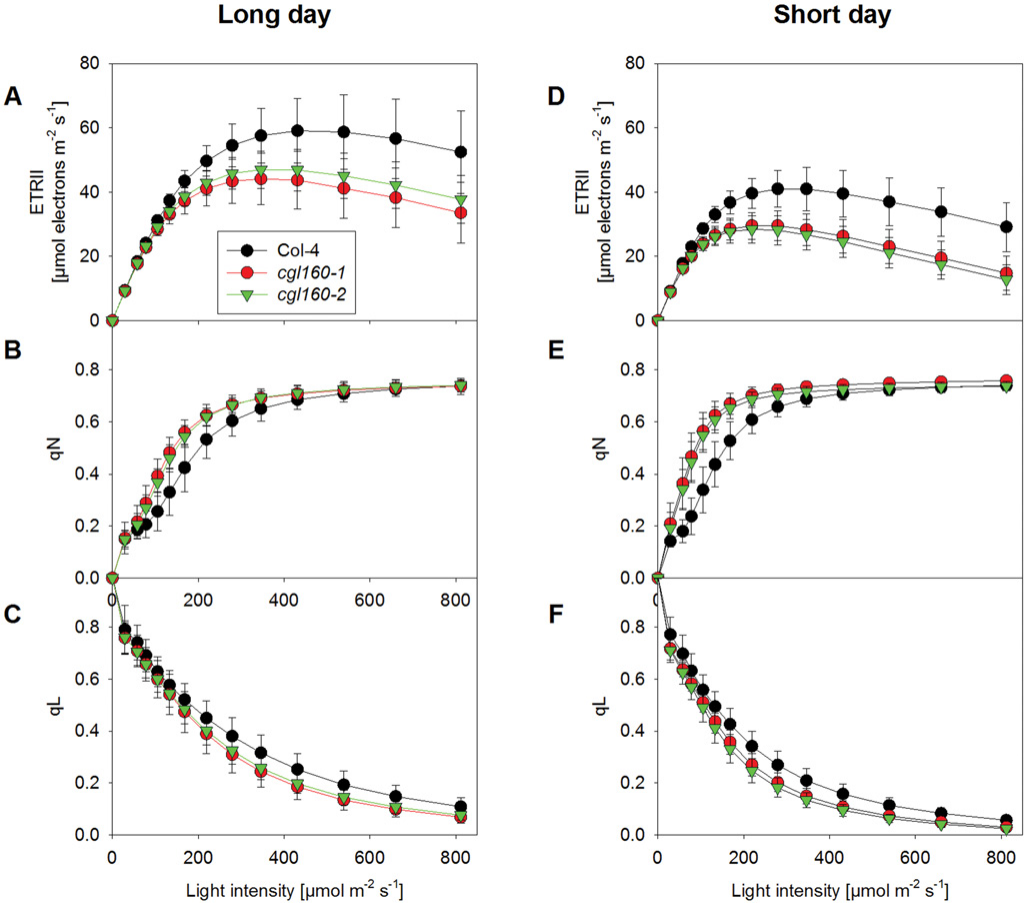
Linear electron transport, non-photochemical quenching (qN) and redox state of the PSII acceptor side are affected in *cgl160* mutants both under long- and short-day conditions. Measurements were done in long (A-C)—and short-day (D-F) adapted plants (16 hours light and 8 hours light, respectively). A and D. Light response curve of linear electron flux as calculated from PSII yield measurements. In both *cgl160* T-DNA insertion lines, linear electron flux capacity is reduced. B and E. Light response curves of non-photochemical quenching (qN). In both T-DNA insertion lines, the induction of qN is shifted towards lower light intensities, but the maximum light-saturated qN is unaltered C and F. Light response curves of the redox state of the PSII acceptor side, determined as qL. When qL is one, Q_A_ is fully oxidized; when qL is zero, Q_A_ is fully reduced. With increasing light intensity, the PSII acceptor side becomes more rapidly reduced in both T-DNA insertion lines.

Decreased activity of chloroplast ATP synthase has been shown to result in reduced proton efflux from the lumen, leading to an increased pmf across the thylakoid membrane already at low actinic light intensities, which in turn can result in a stronger induction of non-photochemical quenching (qN) and down-regulation of linear electron flux due to photosynthetic control [18]. Therefore, we first measured light response curves of linear electron flux (Fig. 5A), non-photochemical quenching (Fig. 5B), and the redox state of the PSII acceptor side (qL) (Fig. 5C) via chlorophyll *a* fluorescence.

**Fig 6:**
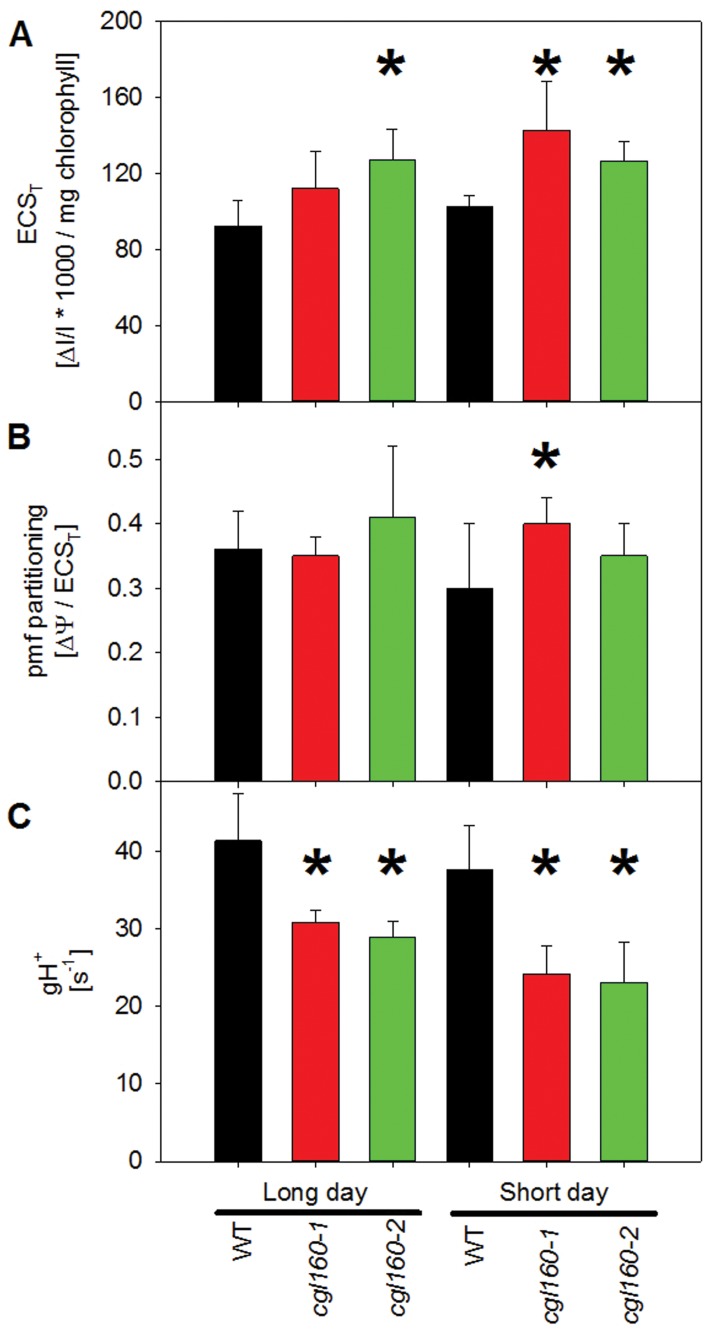
Thylakoid membrane energization, pmf partitioning, and chloroplast ATP synthase activity in Arabidopsis *cgl160* mutant plants. A. The maximum pmf across the thylakoid membrane, as determined from the maximum amplitude of the electrochromic shift signal during a dark-interval relaxation kinetic (ECS_T_), is increased in *cgl160* mutants under long- and short-day conditions. B. pmf partitioning, deduced from the slow relaxation phase of the electrochromic shift signal, is slightly shifted in favor of ΔΨ in Arabidopsis *cgl160* mutant plants under short-day conditions. C. ATP synthase activity (gH^+^), determined from rapid dark-interval relaxation kinetics of the electrochromic shift signal, is significantly decreased in Arabidopsis *cgl160* mutant plants under both long-day and short-day conditions.

Under long-day conditions, there was a tendency towards lower linear electron flux (Fig. 5A) and a slightly stronger induction of qN (Fig. 5B) at low light intensities in both T-DNA insertion lines. Also, the PSII acceptor side was slightly more reduced in low light in the two mutants, relative to the wild type (Fig. 5C). Under short-day conditions, these differences became more pronounced and were statistically significant (Fig. 5D-F; for the capacity of linear electron flux, see also Table 1). Because these changes in chlorophyll *a* fluorescence parameters are compatible with decreased ATP synthase activity and increased lumen acidification, we next directly measured the pmf across the thylakoid membrane (Fig. 6A), its partitioning into ΔΨ and ΔpH (Fig. 6B), and chloroplast ATP synthase activity (Fig. 6C).

ATP synthase activity and thylakoid membrane energization were determined from the dark-interval relaxation kinetic of the electrochromic shift signal (ECS), which were measured after 10 min illumination of leaves with saturating light, so that photosynthesis was in reached steady-state. The total amplitude of the ECS signal measured during a dark interval (ECS_T_) is a measure for the strength of the light-induced pmf across the thylakoid membrane. ECS_T_ was increased in the *cgl160* T-DNA insertion lines, even though this effect was only statistically significant under short-day conditions, indicating an increased pmf across the thylakoid membrane (Fig. 6A). To determine if the increased pmf translates into stronger thylakoid lumen acidification, we determined the pmf partitioning into ΔpH and ΔΨ by measuring the slow phase of the ECS relaxation kinetic, which occurs on a timescale of seconds and is attributable to counter-ion movements across the thylakoid membrane and therefore proportional to the ΔpH component of the pmf. Between 30 and 40% of the total pmf was partitioned into ΔΨ, with the ΔpH accounting for 60 to 70% of total pmf (Fig. 6B). Pmf partitioning was slightly shifted in favor of the ΔΨ component, even though this effect was only significant in the *cgl160-1* line under short-day conditions. Therefore, the increase of the total pmf across the thylakoid membrane does not linearly translate into an increased lumen acidification. Because the rapid decay of the pmf during a dark interval directly reflects the proton efflux rate through the ATP synthase from the thylakoid lumen to the stroma, the reciprocal value of the lifetime of the pmf during the dark interval is a measure of the thylakoid membrane conductivity for protons, gH^+^, which reflects ATP synthase activity. Exemplary dark-interval relaxation kinetics obtained for a wild-type plant and both T-DNA insertion lines under short-day conditions are shown in S3 Fig.

For better comparability of the decay kinetics, the signals were normalized. ATP synthase activity was significantly reduced by about 25 to 40% in the two T-DNA insertion lines under both growth conditions (Fig. 6C).

Next, we quantified the different photosynthetic complexes by difference absorbance spectroscopy in isolated thylakoids, and then normalized the data on a leaf area basis (Table 1). For the accumulation of PSII, the cytochrome *b*_6_ *f* complex, and plastocyanin, no significant differences were observed under both growth conditions. Chlorophyll content and photosynthetic complex accumulation were approximately 20 to 25% lower under short-day conditions than in long-day grown plants (Table 1). PSI contents did not differ significantly between the wild type and the mutants under long-day conditions, but they were slightly, yet significantly, reduced in both mutants under short-day conditions (Table 1). Decreased PSI accumulation has previously been reported in other ATP synthase mutants [18], so this observation is also compatible with a role of CGL160 in ATP synthase accumulation. Finally, to assess changes in antenna distribution between the two photosystems, 77K chlorophyll *a* fluorescence emission spectra between 660 and 800 nm wavelength were measured (S4 Fig.). While under long-day conditions, no clear differences in the 77K fluorescence emission spectra could be observed between the wild type and the two T-DNA insertion lines, under short-day conditions, chlorophyll *a* fluorescence emission from PSI-LHCI at 732 nm wavelength was slightly decreased in both *cgl160* T-DNA insertion lines, in line with the decreased PSI accumulation observed in the mutants.

**Fig 7:**
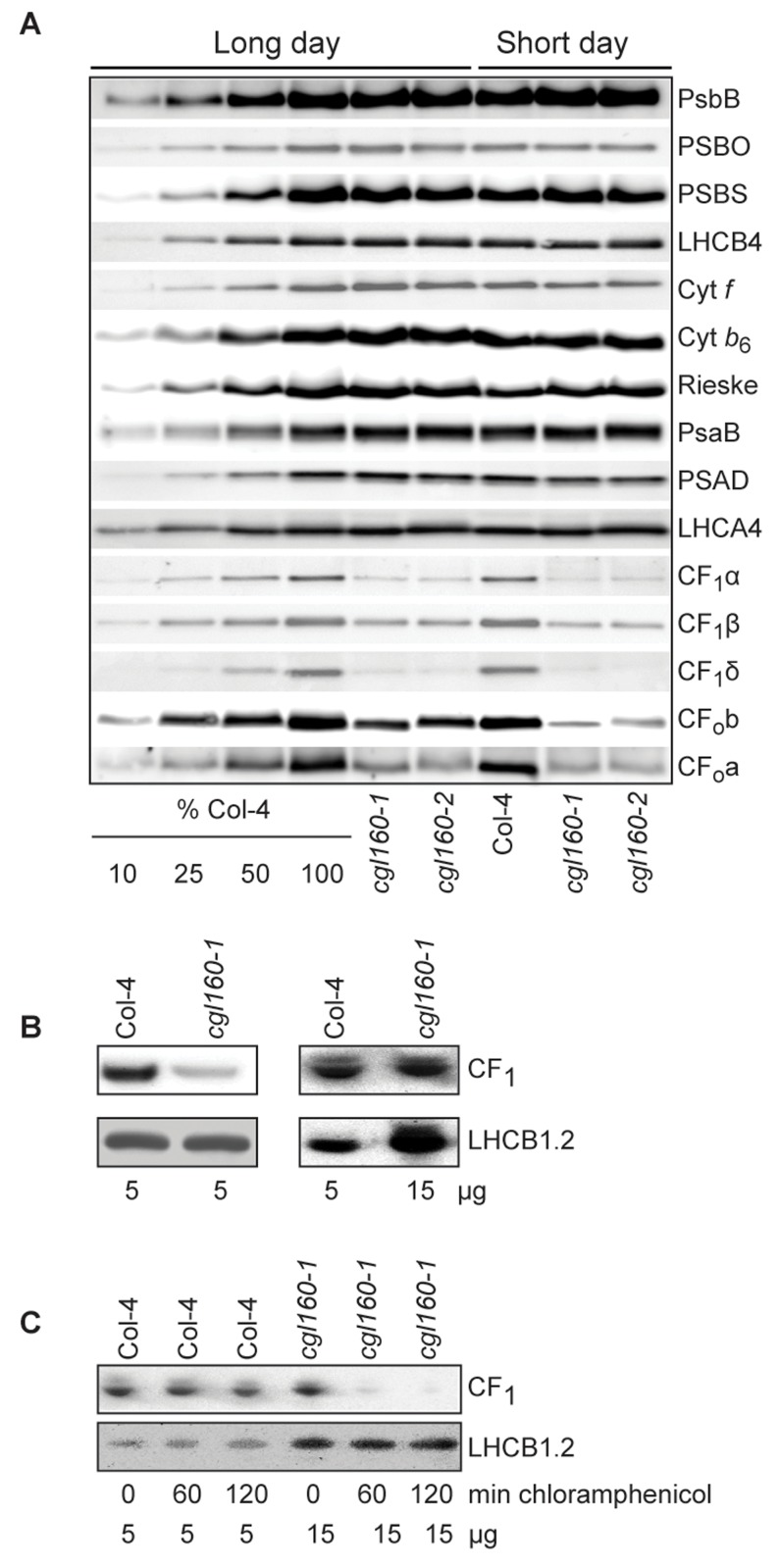
Altered protein accumulation and stability of the chloroplast ATP synthase in the *cgl160* mutant visualized by immunoblotting. A. Immunoblots with antibodies against essential subunits of the photosynthetic protein complexes of wild-type (Col-4) Arabidopsis and the two *cgl160* T-DNA insertion lines grown under long-day and short-day conditions. Isolated thylakoid membranes were used, and equal amounts of chlorophyll were loaded onto the SDS-PAGE gel. For approximate quantification, wild-type samples from long-day plants were diluted to 10%, 25% and 50%, respectively. Accumulation of PSII was probed with antibodies against PsbB and PSBO. Additionally, the PSBS protein involved in NPQ and the minor PSII antenna protein LHCB4 were probed. Accumulation of the cytochrome *b*_6_ *f* complex was probed with antibodies against the essential subunits PetA (cytochrome *f*), PetB (cytochrome *b*_6_), and PETC (Rieske protein). Accumulation of PSI was probed with antibodies against the reaction center subunit PsaB and the stromal ridge subunit PsaD. ATP synthase accumulation was probed with antibodies against the CF_1_ subunits AtpA (CF_1_α), AtpB (CF_1_β) and AtpD (CF_1_δ) and antibodies against the CF_0_ subunits AtpF (CF_0_b) and AtpI (CF_0_a). B. Loading difference estimation for immunoblotting CF_1_ between wild type and *cgl160-1*. To obtain similar immunoblotting signal three times more (15 μg protein) was needed for *cgl160-1* compared to wild type (5 μg protein). C. Maintenance of CF_1_ was measured by incubating leaves from wild type and *cgl160-1* in solution containing the plastid protein synthesis inhibitor chloramphenicol for the indicated time points. Protein extract was isolated and separated by SDS-PAGE, immunoblotted and probed with specific antibodies against CF_1_ and LHCB2.1. Three times more protein was loaded from the mutant to obtain equal level of CF_1_ immunoblotting signal, as specified in B.

The photosynthesis data are compatible with a role of CGL160 in ATP synthase accumulation, in that a decreased content of ATP synthase results in a reduced enzyme activity. Alternatively, they could be explained by a decreased activation of the enzyme, due to changes in its post-translational modification. To distinguish between these scenarios, we quantified accumulation of different ATP synthase subunits by immunoblots (Fig. 7A).

Equal amounts of chlorophyll were loaded onto the gel for separation of proteins by SDS-PAGE, and a dilution series of wild-type thylakoid membranes from long-day conditions was used to quantify changes in protein abundances. The abundances of the CF_1_-subunits α, β and δ were clearly reduced in both *cgl160* mutants, down to less than 25% of wild-type levels. The reduction was clearly more pronounced under short-day than under long-day conditions. Similar observations were obtained for the two CF_0_-subunits b or AtpF and a or AtpI, which again displayed more severe reductions under short-day than under long-day conditions. Obviously, ATP synthase contents are much more reduced than ATP synthase activity. This agrees well with data by [18], who showed that only a more than 50% reduction in ATP synthase contents resulted in clear effects on enzyme activity. Besides ATP synthase subunits, the accumulation of the essential PsbB subunit and the 33kDa subunit of the oxygen evolving complex (PSBO) of PSII, of the photoprotective PSBS protein, of a minor light harvesting complex II subunit (LHCB4), of the essential cytochrome *b*_6_ *f* complex subunits cytochrome *f*, cytochrome *b*_6_ and the Rieske protein, and the essential PSI subunits PsaB and PSAD and the light harvesting complex I subunits (LHCA4) were also probed by immunoblotting. Neither under long-day nor short-day conditions, did we observe major differences between the wild type and the T-DNA insertion mutants (Fig. 7A). Because the decreased complex contents per leaf area observed under short-day conditions are largely attributable to the lower total chlorophyll amount (Table 1), the immunoblot data are well in agreement with the spectroscopic data (Table 1). To verify that the decreased accumulation of ATP synthase subunits in the mutant plants was because of a lack of CGL160 we complemented the *cgl160-1* line with the wild type gene. This restored the accumulation of the ATP synthase subunits to wild type levels (S5 Fig.).

To investigate the stability of the ATP synthase proteins, leaves from wild type and *cgl160-1* were incubated in a solution containing the plastid protein synthesis inhibitor chloramphenicol for the indicated time points (Fig. 7C). As chloramphenicol inhibits chloroplast translation no new proteins are added to the membrane and thus the status quo of existing proteins can be visualized, which is an assay for the half-life of the protein. Because the *cgl160* mutant plants accumulate less ATP synthase compared to wild type, we loaded three times more protein for the mutants than for the wild type (Fig. 7B). By using immunoblotting on total plant extracts it was strikingly clear that after 120 min incubation time, wild-type leaves retained ATP synthase while in the mutant after 120 min, the ATP synthase amount was strongly reduced (Fig. 7C). This was regardless of the fact that three times more protein was loaded as compared to wild type. As a loading control LHCB1.2 was used and the abundance of this protein stayed unchanged in both wild-type and mutant leaves (Fig. 7C).

### CGL160 interacts with CF_1_-containing assembly intermediates of chloroplast ATP synthase

**Fig 8:**
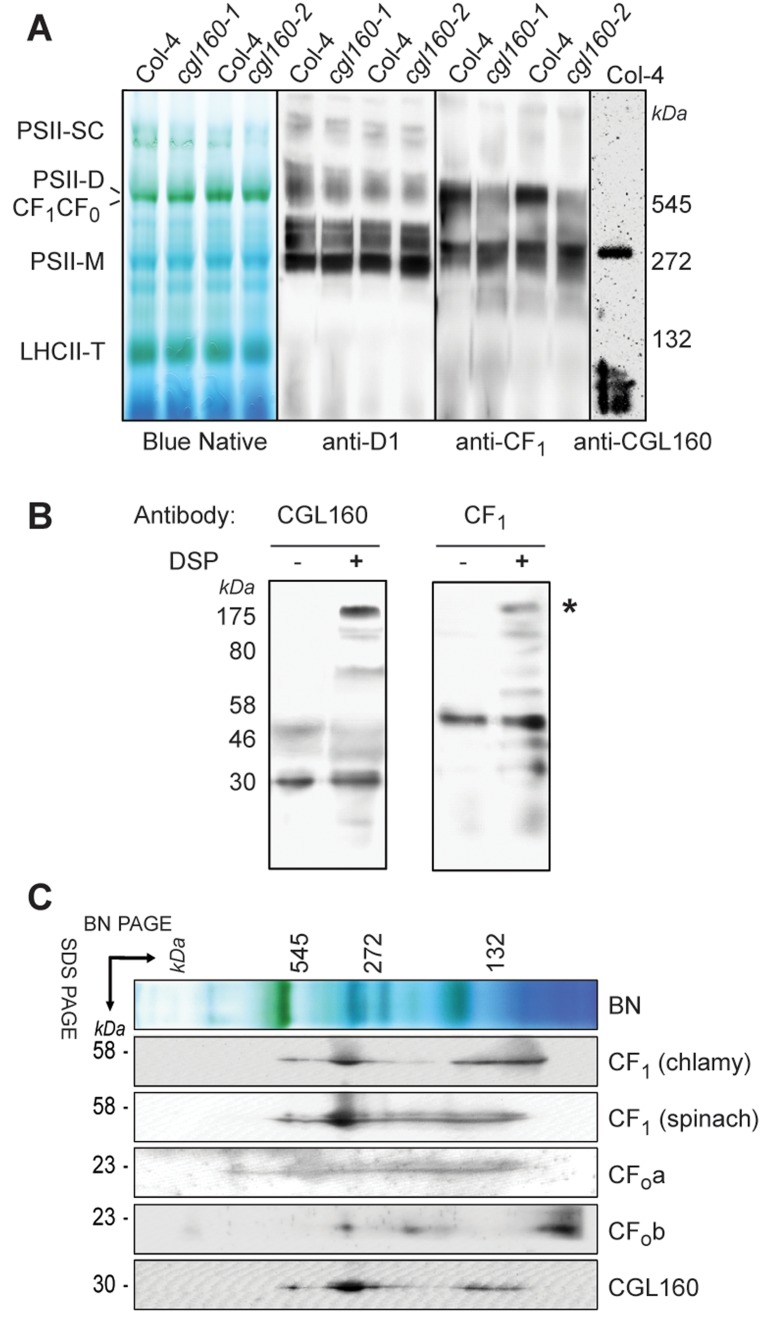
2D native / SDS-PAGE gel immunoblotting and crosslinking indicate interaction between CGL160 and the chloroplast ATP synthase complex. A. Blue-native gel electrophoresis analysis of thylakoidal protein complexes in wild type and mutant plants. PSII-SC photosystem II supercomplex, PSII-D photosystem II dimer, PSII-M photosystem II monomer, LHCII-T light harvesting complex II trimer. The Blue-native gel was used for immunoblotting against D1, CF_1_ and CGL160 antibodies as indicated. B. Solubilized and cross-linked thylakoid membranes were separated by SDS-PAGE and probed with specific antibodies against CGL160 and CF_1_. C. Detection of CGL160 and ATP synthase subcomplexes by immunoblot analyses of 2D BN/SDS gels as in panel A, employing antibodies specific for CGL160 and individual ATPase subunits as indicated in the figure.

To test if CGL160 might play a role in the expression of the six plastid-encoded subunits of chloroplast ATP synthase, we tested steady-state mRNA abundances of *atpA*, *atpB*, *atpE*, *atpF*, *atpH* and *atpI* in wild-type and *cgl160-1* plants by qPCR (S2 Table). None of the mRNAs was significantly reduced, but the expression of *atpE* and *atpH* was significantly increased in *cgl160*-1, possibly in a feedback response to the decreased ATP synthase accumulation. Furthermore, it was recently demonstrated that translation of the major thylakoid protein complexes showed no difference between the wild type and plants lacking CGL160 [25]. To determine whether CGL160 interacts with any major complex in the thylakoid membrane, immunoblots of BN-PAGE separated thylakoid membranes from Arabidopsis were probed with the CGL160 antibody (Fig. 8A).

The antibody detected one large complex at around ~300 kDa. This CGL160-containing complex co-migrated with one major CF_1_-containing complex, detected with an antibody raised against the entire CF_1_ part of ATP synthase [43], and this corresponds indeed to the CF_1_ portion (Fig. 8A). The other major ATP synthase CF_1_-CF_0_ band at around 500 kDa did not include any CGL160, indicating that CGL160 is not a stable component of the mature ATP synthase complex but rather works as a stability factor specific for the CF_1_ stroma facing portion. To further validate the interaction between CGL160 and ATP synthase, we used the cross-linker DSP (Dithiobis-succinimidyl propionate), which is reactive toward amino groups. This cross-linker has earlier been used in nearest neighbor analyses of isolated photosynthetic complexes [37, 44]. When thylakoid membranes were treated with DSP, the CGL160 protein became partly cross-linked in a higher molecular weight / mass complex around 170 kDa (Fig. 8B). The same crosslinked and non crosslinked control samples were tested by ATP synthase CF_1_ antibody and showed a signal at the same high molecular weight complex (Fig. 8B), indicating that both CGL160 and CF_1_ proteins interact in this complex. To further visualize the co-migration of CGL160 and ATP synthase, we performed BN-PAGE analyses followed by separation into their subunits in the second dimension by electrophoresis on SDS-PAGE gels, and blotted onto PVDF membranes (Fig. 8C). Two different CF_1_ antibodies were used and showed a very reproducible spot at around 300 kDa, and as expected CGL160 showed one major signal at this spot as well. AtpI and AtpF (subunits a and b) displayed several signals but did not line up with CGL160 and CF_1_ (Fig. 8C).

## Discussion

In this manuscript, we present the functional characterization of CGL160, a new auxiliary protein supporting the assembly of chloroplast ATP synthase. CGL160 is conserved in all organisms performing oxygenic photosynthesis. In cyanobacteria, *cgl160* is part of an operon encoding seven ATP synthase subunits, some of these belonging to the membrane-intrinsic F_0_ part of the enzyme, and some belonging to the catalytic F_1_ part (Fig. 1A). In Arabidopsis, the CGL160 homologue At2g31040 is nucleus-encoded and imported into the chloroplasts. Physiological data strongly suggest a role of CGL160 in either the biogenesis or stabilization of ATP synthase. Analyses of one T-DNA insertion mutant (*cgl160-1*), which is completely devoid of the CGL160 protein (Fig. 2B), and a second mutant (*cgl160-2*), which suffers from an 80% reduction in CGL160 protein content (Fig. 2C), revealed identical photosynthetic defects: Under long-day growth conditions, both T-DNA insertion lines hardly show a growth retardation, while under short-day and especially under fluctuating light conditions, the phenotype is more pronounced, and biomass accumulation is reduced (Fig. 4A-C, S2 Fig.). Accumulation of chloroplast ATP synthase is decreased to less than 25% of wild-type levels in both T-DNA insertion mutants under constant light conditions (Fig. 7A). The fact that plants deficient in CGL160 still accumulate chloroplast ATP synthase, but to much lower levels than the wild type, shows that CGL160 is non-essential for ATP synthase biogenesis, but supports this process.

The approximately fourfold reduction in ATP synthase observed in both T-DNA insertion lines under constant long-day conditions has only moderate effects on plant growth and photosynthesis, because ATP synthase activity is much less reduced than ATP synthase content (Fig. 6C). This observation is similar to what has already been reported for tobacco ATP synthase mutants [18]. In wild-type tobacco, a large amount of ATP synthase is inactive, possibly due to post-translational modifications such as phosphorylation of AtpB [45, 46]. Only a more than 50% reduction in ATP synthase content resulted in a significant decrease of ATP synthase activity. Here, we demonstrate that in Arabidopsis, a similar excess amount of ATP synthase seems to be present, in that a more than 75% reduction of ATP synthase content only results in a 25% to 40% reduction of enzyme activity under long-day and short-day conditions, respectively. Besides chloroplast ATP synthase, the accumulation of the other photosynthetic complexes is unaltered under long-day conditions, while under short-day conditions, accumulation of PSI is slightly decreased on a leaf area basis (Table 1), in line with a previous report for ATP synthase-deficient tobacco transformants [18].

The antenna structure of the photosystems is only marginally affected, as shown by 77K chlorophyll *a* fluorescence emission spectra (S4 Fig.). As a consequence of the decreased ATP synthase activity, the pmf across the thylakoid membrane is increased (Fig. 6A). Because the pmf partitioning tends to be shifted in favor of the ΔΨ component (Fig. 6B), the thylakoid lumen is only slightly more acidic than in the wild type. Therefore, lumen pH-dependent processes such as the down-regulation of linear electron flux by photosynthetic control (Fig. 5A) and the induction of non-photochemical quenching (qN) (Fig. 5B) are only moderately shifted towards lower light intensities. A similar but slightly more pronounced shift in pmf partitioning in favor of the ΔΨ component has been observed in tobacco transformants with reduced ATP synthase contents [18], indicating that this may allow plants to somewhat compensate for an increased total pmf across the thylakoid membrane. An increased pmf partitioning into ΔΨ would allow plants to partly reduce detrimental effects of a reduced ATP synthase accumulation and an increased pmf across the thylakoid membrane, such as a lower quantum efficiency of CO_2_ fixation under light-limited conditions, because the induction of non-photochemical quenching is exclusively controlled by the ΔpH component of the pmf. However, it is still matter of debate how and to which extent higher plants can control their pmf partitioning [47]. Recently, several putative ion channels and antiporters in the thylakoid membrane have been identified, which might control the pmf partitioning [48, 49]. However, the fact that both in the *cgl160* mutants and in the ATP synthase deficient tobacco transformants, only a small change in pmf partitioning was observed, so that still, non-photochemical quenching was induced at lower actinic light intensities, may suggest that the capacity of plants to adjust pmf partitioning is more limited than assumed.

CGL160 seems to act on a post-translational level on ATP synthase accumulation: By using quantitative PCR, we found no strong differences in the mRNA abundances of chloroplast ATP synthase subunits between mutant and wild-type plants (S2 Table). A direct interaction of CGL160 with ATP synthase subunits is also supported by the observation that CGL160 is stably associated with the thylakoid membranes (Fig. 3C) and exclusively found in the non-appressed stroma lamellae (Fig. 3B). This localization is similar to that of ATP synthase itself, because the bulky CF_1_ sterically excludes ATP synthase from appressed membranes [50, 51]. Furthermore, crosslinking studies indicate that CGL160 co-migrates with two ATP synthase subcomplexes of 300 kDa molecular mass and 170 kDa molecular mass in BN-gels (Fig. 8). Both subcomplexes contain subunits of CF_1_, and at least the 300 kDa subcomplex also seems to contain a small amount of the CF_0_ subunit b (AtpF). However, only a very minor fraction of CGL160 is associated with the fully assembled ATP synthase complex of approximately 500 kDa molecular mass (Fig. 8A and 8B). The majority of CGL160 associates with sub-complexes of ATP synthase, indicating a role in mediating the assembly of ATP synthase, or at least in stabilizing subcomplexes during ATP synthase biogenesis, because in *cgl160* mutant plants, the half-life of ATP synthase subunits was severely decreased (Fig. 7C). These subcomplexes definitely contain CF_1_ subunits, while for CF_0_ subunits, the results are less clear, strongly suggesting a preferential interaction of CGL160 with partly and / or fully assembled CF_1_.

A specific role of CGL160 in ATP synthase assembly is also supported by phylogenetic data: CGL160 bears a moderate similarity to the prokaryotic UncI protein [25]. UncI supports the c-ring formation of the F_0_F_1_-ATP synthase in the Gram-negative bacterium *Propionigenium modestum* [52, 53]. There, UncI directly interact with the subunits of the c-ring and might support the c-ring formation. A similar function in CF_0_ formation was recently postulated for CGL160 in Arabidopsis as well [25]. On the other hand, our data point more towards an interaction of CGL160 with CF_1_, possibly mediating the assembly of CF_0_ and CF_1_ with each other. The suggestion of a specific role of CGL160 in c-ring formation in Arabidopsis by [25] was based on the observations that:

- CF_0_ subunits were more strongly depleted than of CF_1_ subunits in isolated thylakoids;
- The population of free, unassembled c-ring subunits was more than 10-fold increased in *cgl160* mutants, relative to the wild type, while the abundance of free forms of the other subunits was less strongly increased;
- CGL160 directly interacts with AtpH (subunit c) in a split-ubiquitin assay.

Most of these observations somewhat contradict our data: We do not observe a selective loss of CF_0_ subunits in our immunoblots (Fig. 7A). It is difficult to explain a selective loss of CF_0_ subunits when immunoblots are performed with isolated thylakoids, because even if CF_1_ subunits or an entire CF_1_ subcomplex would quantitatively accumulate in the absence of CF_0_, they would not be stably associated with the thylakoid membrane, and therefore should be lost during the thylakoid isolation procedure. Also, our crosslinking data show that most of CGL160 is associated with ATP synthase subcomplexes mainly containing CF_1_ subunits. Therefore, we suggest that at least in addition to a role in c-ring formation, CGL160 also supports the assembly of CF_1_, or might at least stabilize CF_1_ until its association with CF_0_. This scenario is well in line with the recently proposed assembly pathway of chloroplast ATP synthase: Mainly based on knowledge of ATP synthase assembly in eubacteria, but also on data for the green alga *C*. *reinhardtii*, [14] suggested that first, a subcomplex of CF_1_ comprising α_3_β_3_γε is assembled in the stroma, which then interacts with the c_14_ ring in the thylakoid membrane. Afterwards, assembly progresses through the binding of this assembly intermediate to a thylakoid-intrinsic abb′ stalk sub-complex, whose assembly is still unknown. This is followed by the final stabilization of the abb′c_14_ α_3_β_3_γε complex by the binding of the δ subunit, which also induces a conformational change in ATP synthase, so that it is capable of proton translocation through CF_0_. In this pathway, the establishment of the interaction of the stromal subcomplex α_3_β_3_γε with the c_14_ ring is a crucial step, and because our data and the data in [25] suggest an interaction of CGL160 with both sub-complexes, this auxiliary protein might stabilize both the c_14_ ring and α_3_β_3_γε in conformations, which then allow their interaction and the formation of the c_14_α_3_β_3_γε assembly intermediate.

In summary, all our observations confirm a specific function of CGL160 in the assembly of chloroplast ATP synthase, either via directly mediating an assembly step of the complex, or via the stabilization of assembly intermediates. Therefore, after Alb4 [54], CGL160 is the second auxiliary protein functioning in chloroplast ATP synthase assembly and / or stability described so far. Alb4 has been suggested to support the attachment of CF_1_ to CF_0_ [54] and therefore might function in the same process as CGL160. This again emphasizes that the formation of the c_14_α_3_β_3_γε assembly intermediate might be a highly critical step of ATP synthase biogenesis.

Two auxiliary proteins for ATP synthase assembly in higher plants is a remarkably low number, because the biogenesis of the other large multiprotein complexes of photosynthesis is supported by a much larger number of auxiliary proteins (recently reviewed by [14]). Their functions range from supporting the co-translational membrane insertion of the chloroplast-encoded proteins, attaching the redox-active cofactors and specific pigments to the nascent polypeptide chains to the processing of subunits, the stabilization of assembly intermediates and the attachment of peripheral subunits to the mediation of protein-protein interactions. In the case of PSII, more than 20 different auxiliary proteins supporting its assembly process have been identified to date [19, 22], and also for PSI and the cytochrome b_6_ *f* complex, more than 10 auxiliary proteins are known [14, 20, 55]. Because ATP synthase neither contains redox-active cofactors nor pigments [56], its assembly might be much less complicated than that of the photosystems and the cytochrome *b*_6_ *f* complex, and a small number of auxiliary proteins might be sufficient. On the other hand, it is possible that additional auxiliary proteins involved in ATP synthase assembly have not been identified until now. Furthermore, after Alb4 in bacteria and mitochondria, a few additional proteins supporting the biogenesis of CF_1_-CF_0_-ATP synthases have been identified. In yeast mitochondria, also the Atp25 protein supports c-ring formation; it also stabilizes the mitochondrial *atp9p* mRNA encoding the c-subunit [57]. Also Atp10 [58, 59] and Atp23 [60] have been implicated in F_0_ assembly in mitochondria. The assembly of F_1_ in yeast mitochondria is supported by the auxiliary proteins Atp11 and Atp12, which interact with the α- and β-subunit [61]. Therefore, in yeast mitochondria, at least five additional proteins support ATP synthase biogenesis, and additional auxiliary factors for chloroplast ATP synthase assembly are likely to be identified in the future (reviewed by [14.]).

## Supporting Information

S1 Fig Phylogenetic tree of sequenced cyanobacterial strains. A. The phylogenetic tree was re-drawn based on [62]. Cyanobacteria that are highlighted in red represent the ones that were used to show the ATP synthase operon arrangement in Fig. 1A.
(TIF)

S2 Fig Plant weight of wild type (Col-4) and *cgl160-1* plants. A. Shoot biomass of Col-4 (white bars) and *cgl160-1* (grey bars) plants growing under long day conditions (16 h light, 8 h dark) at 120 μmol photons m^-2^ s^-1^. B. Shoot biomass of Col-4 (white bars) and *cgl160-1* (grey bars) plants growing under fluctuating light conditions (5 min 120 μmol m^-2^ s^-1^ followed by 5 min 20 μmol m^-2^ s^-1^ changing every 5 min for 16 hours light and then 8 hours dark). Bars represent mean values ± SE, n = 5.
(PNG)

S3 Fig ECS decay kinetics in wild type Arabidopsis Col-4 plants and the two *cgl160* T-DNA insertion mutants grown under short-day conditions. For better comparability of the decay kinetics, the signal was normalized. The maximum light-saturated ECS is set to one, and the fully decayed signal is normalized to zero.
(JPG)

S4 Fig 77K chlorophyll *a* fluorescence emission spectra. The 77K chlorophyll *a* fluorescence emission spectra showing virtually unaltered antenna distribution between both photosystems in the wild type (dark line), and the *cgl160-1* (red) and *cgl160-2* (green) under long-day (A) and short-day conditions (B).
(JPG)

S5 Fig Complementation of the *cgl160-1* mutant. Immunodetection of CGL160 and ATP synthase accumulation in chloroplasts isolated from wild type (Col-4) and complemented *cgl160-1C* plants. Antibodies as described for Fig. 2 and Fig. 7.
(TIF)

S1 Table Primers. List of the primers used for PCR experiments.
(PNG)

S2 Table qRT-PCR. qRT-PCR on chloroplast ATPase synthase subunits shows no major difference between wild type and *cgl160* mutant plants. Each plant genotype was analyzed three times in biological replicates in three technical replicates.
(PNG)
