# Supplementary figures and images for: The Thylakoid Membrane Protein CGL160 Supports CF1CF0 ATP Synthase Accumulation in Arabidopsis thaliana
Source: PLoS One. 2015 Apr 2;10(4):e0121658. doi: 10.1371/journal.pone.0121658 (PMC4383579; doi:10.1371/journal.pone.0121658)

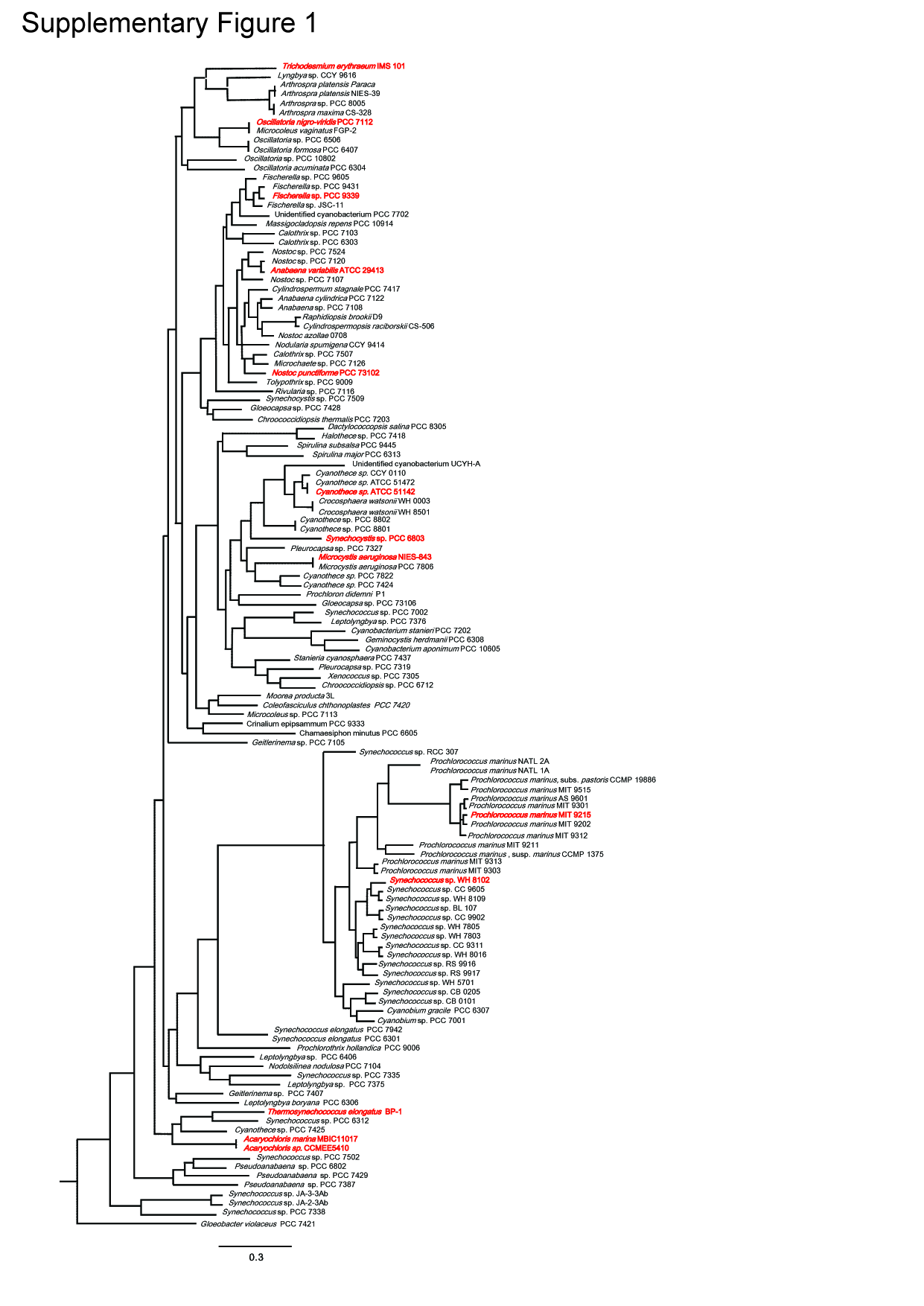

Supplement: S1 Fig — A. The phylogenetic tree was re-drawn based on [62]. Cyanobacteria that are highlighted in red represent the ones that were used to show the ATP synthase operon arrangement in Fig. 1A. (TIF) [file pone.0121658.s001.tif]

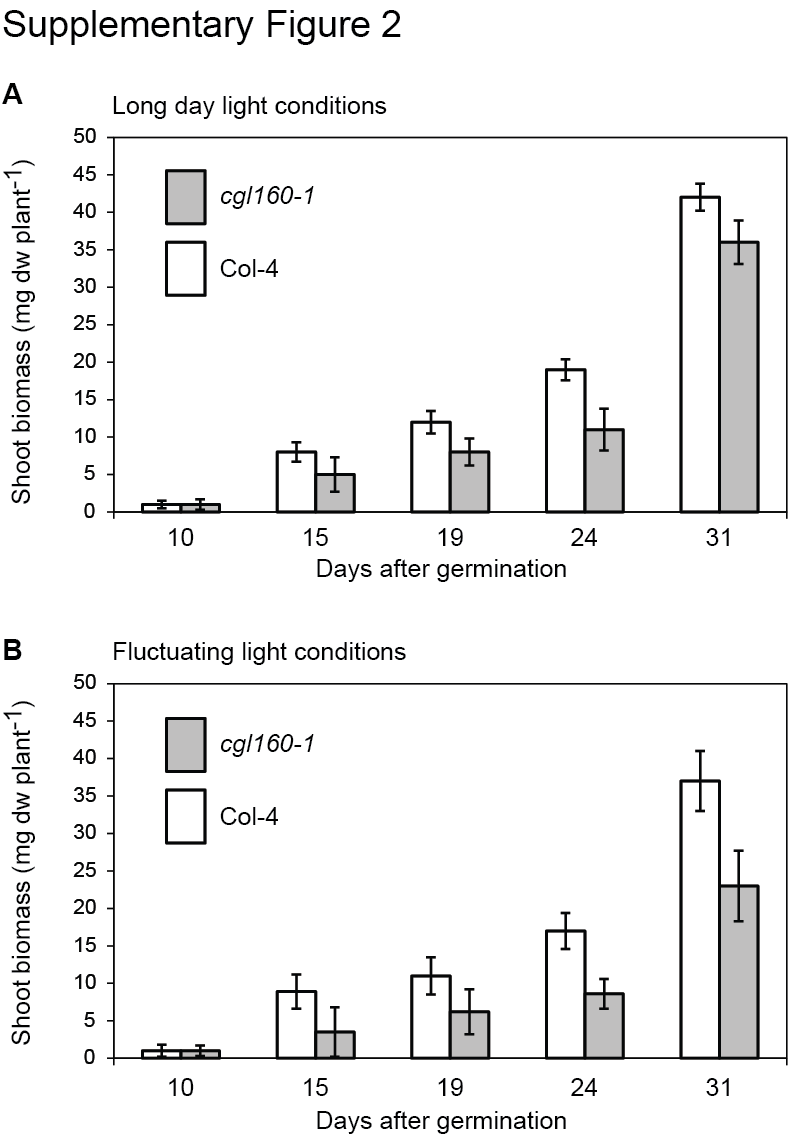

Supplement: S2 Fig — A. Shoot biomass of Col-4 (white bars) and cgl160-1 (grey bars) plants growing under long day conditions (16 h light, 8 h dark) at 120 μmol photons m-2 s-1. B. Shoot biomass of Col-4 (white bars) and cgl160-1 (grey bars) plants growing under fluctuating light conditions (5 min 120 μmol m-2 s-1 followed by 5 min 20 μmol m-2 s-1 changing every 5 min for 16 hours light and then 8 hours dark). Bars represent mean values ± SE, n = 5. (PNG) [file pone.0121658.s002.png]

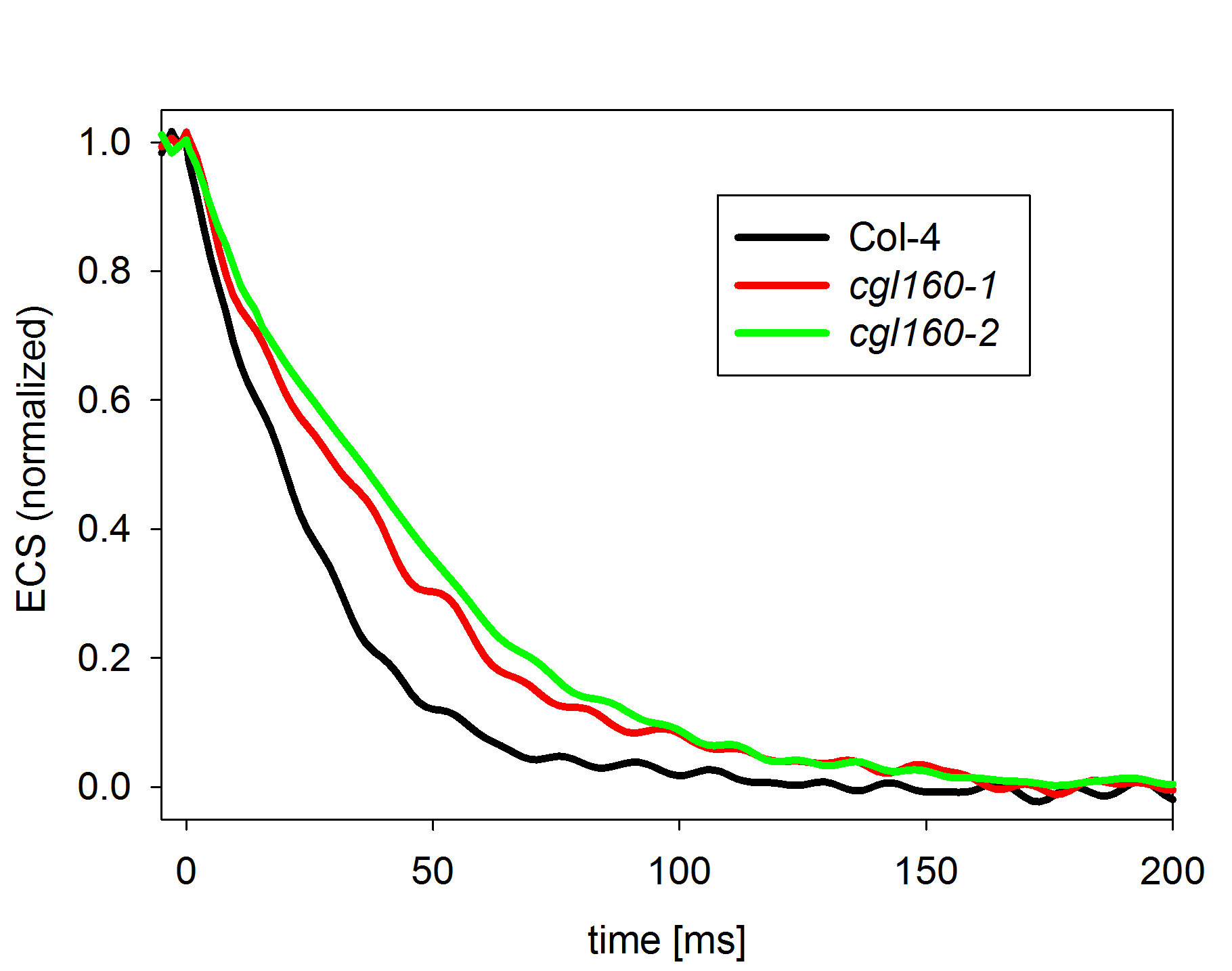

Supplement: S3 Fig — For better comparability of the decay kinetics, the signal was normalized. The maximum light-saturated ECS is set to one, and the fully decayed signal is normalized to zero. (JPG) [file pone.0121658.s003.JPG]

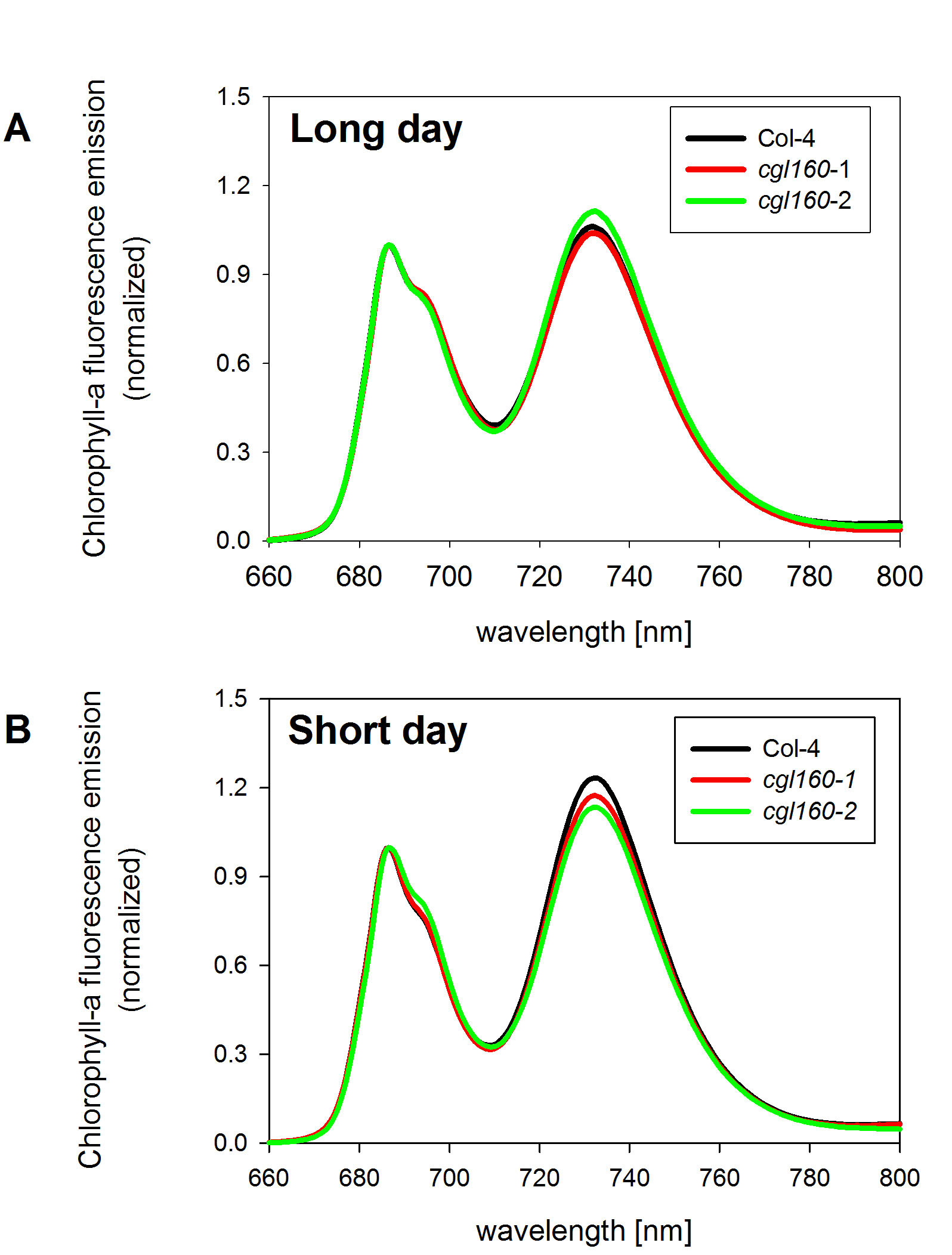

Supplement: S4 Fig — The 77K chlorophyll a fluorescence emission spectra showing virtually unaltered antenna distribution between both photosystems in the wild type (dark line), and the cgl160-1 (red) and cgl160-2 (green) under long-day (A) and short-day conditions (B). (JPG) [file pone.0121658.s004.jpg]

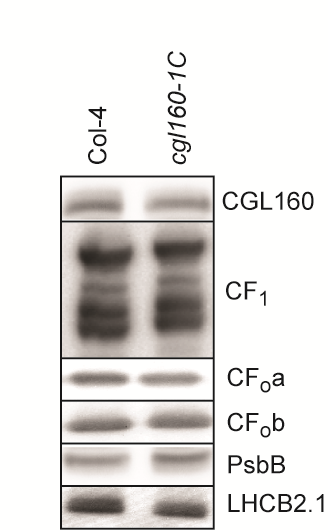

Supplement: S5 Fig — Immunodetection of CGL160 and ATP synthase accumulation in chloroplasts isolated from wild type (Col-4) and complemented cgl160-1C plants. Antibodies as described for Fig. 2 and Fig. 7. (TIF) [file pone.0121658.s005.tif]

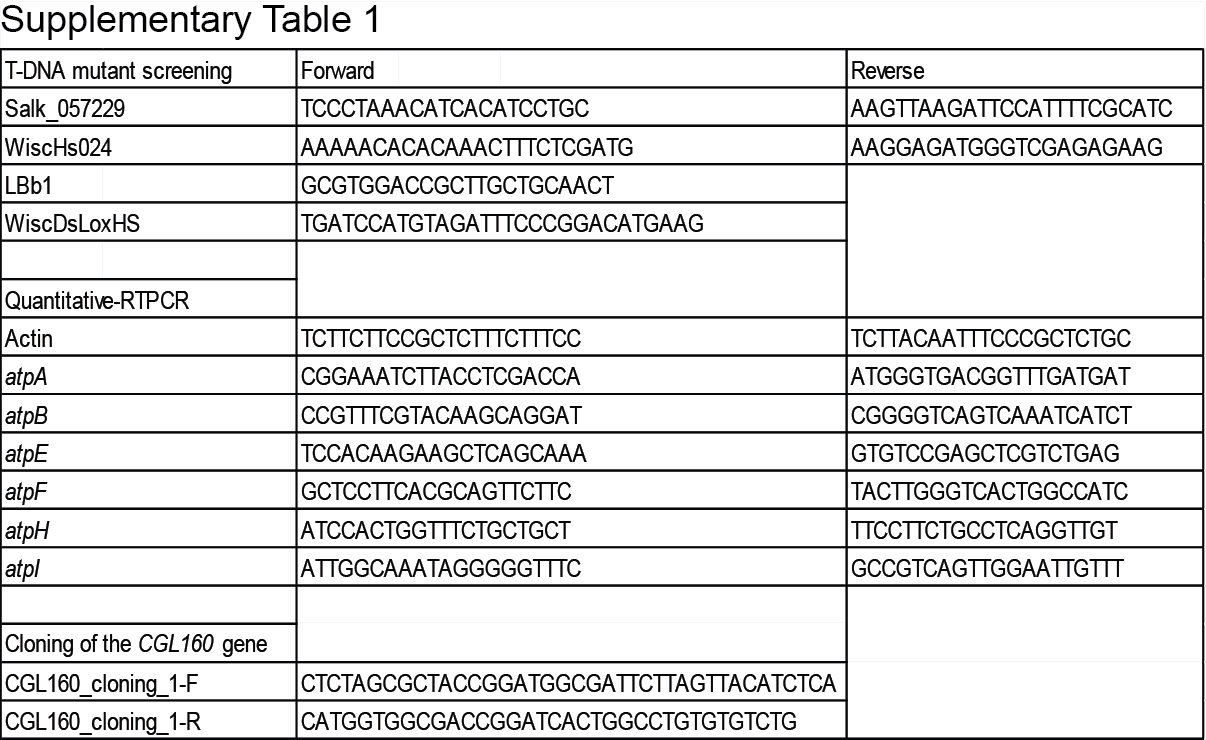

Supplement: S1 Table — List of the primers used for PCR experiments. (PNG) [file pone.0121658.s006.png]

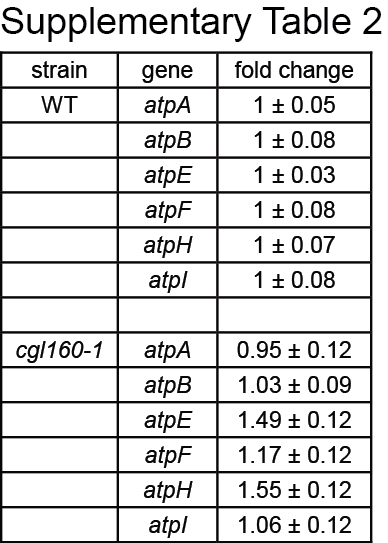

Supplement: S2 Table — qRT-PCR on chloroplast ATPase synthase subunits shows no major difference between wild type and cgl160 mutant plants. Each plant genotype was analyzed three times in biological replicates in three technical replicates. (PNG) [file pone.0121658.s007.png]
